# Phylogeny of Kinorhyncha Based on Morphology and Two Molecular Loci

**DOI:** 10.1371/journal.pone.0133440

**Published:** 2015-07-22

**Authors:** Martin V. Sørensen, Matteo Dal Zotto, Hyun Soo Rho, Maria Herranz, Nuria Sánchez, Fernando Pardos, Hiroshi Yamasaki

**Affiliations:** 1 Section of GeoGenetics, Natural History Museum of Denmark, University of Copenhagen, Copenhagen, Denmark; 2 Department of Life Sciences, University of Modena and Reggio Emilia, Modena, Italy; 3 Dokdo Research Center, Korean Institute of Ocean Science and Technology, Uljin, Korea; 4 Department of Zoology and Anthropology, Universidad Complutense de Madrid, Madrid, Spain; 5 Department of Chemistry, Biology & Marine Science, University of the Ryukyus, Okinawa, Japan; Sars International Centre for Marine Molecular Biology, NORWAY

## Abstract

The phylogeny of Kinorhyncha was analyzed using morphology and the molecular loci 18S rRNA and 28S rRNA. The different datasets were analyzed separately and in combination, using maximum likelihood and Bayesian Inference. Bayesian inference of molecular sequence data in combination with morphology supported the division of Kinorhyncha into two major clades: Cyclorhagida comb. nov. and Allomalorhagida nom. nov. The latter clade represents a new kinorhynch class, and accommodates *Dracoderes*, *Franciscideres*, a yet undescribed genus which is closely related with *Franciscideres*, and the traditional homalorhagid genera. Homalorhagid monophyly was not supported by any analyses with molecular sequence data included. Analysis of the combined molecular and morphological data furthermore supported a cyclorhagid clade which included all traditional cyclorhagid taxa, except *Dracoderes* that no longer should be considered a cyclorhagid genus. Accordingly, Cyclorhagida is divided into three main lineages: Echinoderidae, Campyloderidae, and a large clade, ‘Kentrorhagata’, which except for species of *Campyloderes*, includes all species with a midterminal spine present in adult individuals. Maximum likelihood analysis of the combined datasets produced a rather unresolved tree that was not regarded in the following discussion. Results of the analyses with only molecular sequence data included were incongruent at different points. However, common for all analyses was the support of several major clades, i.e., Campyloderidae, Kentrorhagata, Echinoderidae, Dracoderidae, Pycnophyidae, and a clade with *Paracentrophyes *+ New Genus and *Franciscideres *(in those analyses where the latter was included). All molecular analyses including 18S rRNA sequence data furthermore supported monophyly of Allomalorhagida. Cyclorhagid monophyly was only supported in analyses of combined 18S rRNA and 28S rRNA (both ML and BI), and only in a restricted dataset where taxa with incomplete information from 28S rRNA had been omitted. Analysis of the morphological data produced results that were similar with those from the combined molecular and morphological analysis. E.g., the morphological data also supported exclusion of *Dracoderes* from Cyclorhagida. The main differences between the morphological analysis and analyses based on the combined datasets include: 1) Homalorhagida appears as monophyletic in the morphological tree only, 2) the morphological analyses position *Franciscideres* and the new genus within Cyclorhagida near Zelinkaderidae and Cateriidae, whereas analyses including molecular data place the two genera inside Allomalorhagida, and 3) species of *Campyloderes* appear in a basal trichotomy within Kentrorhagata in the morphological tree, whereas analysis of the combined datasets places species of *Campyloderes* as a sister clade to Echinoderidae and Kentrorhagata.

## Introduction

The metazoan phylum Kinorhyncha was discovered by the French naturalist Felix Dujardin [1]. In 1841 he collected marine invertebrates at Saint Malo on the north coast of Bretagne in France, and found what later turned out to be the kinorhynch *Echinoderes dujardinii* Claparède, 1863 [2]. Today, more than 170 years after Dujardin’s discovery, the phylum accommodates about 222 described species, distributed on 23 genera (see [3], [4] for the most recent reviews of kinorhynch classification and taxonomy). The newly described genus, *Mixtophyes* Sánchez, Pardos & Sørensen, 2014 (see [5]) was described recently and has not yet been included in any lists or reviews of general kinorhynch taxonomy.

Today, kinorhynchs are considered part of the Ecdysozoa. This is supported by morphological [6], as well as molecular evidence [7], [8], [9]. Usually kinorhynchs are accommodated within the group Scalidophora [6], [10], together with Priapulida and Loricifera, and even though scalidophoran monophyly has been questioned in regard to the loriciferans [11], [12], we can at least consider Kinorhyncha and Priapulida as closely related and potential sister groups.

Whereas kinorhynchs have been represented in numerous general metazoan phylogenies, surprisingly few attempts have been made to understand the phylogenetic relationships within Kinorhyncha. Until very recently, kinorhynch classification was based on systematic hierarchies proposed initially by Zelinka [13] and later modified and updated by Higgins [14], [15] and Adrianov and Malakhov [16], [17]. Obviously, these classifications were based on very comprehensive knowledge and insights in kinorhynch taxonomy and morphology, but they suffered by the fact that they were not based on Hennigian thinking, and for instance, did not discriminate between apomorphic and plesiomorphic character conditions. Another shortcoming was that the systems were not based on objective numerical analysis of empiric data, but were merely a result of phenotypic “by hand” classification. Notwithstanding these problems, present days’ kinorhynch classification follows to a great extent Higgins [15] and Adrianov and Malakhov [17]. The two systems differ at some minor points (mostly regarding the interrelationships of the families), but basically they both agree to divide Kinorhyncha into the two main groups: Cyclorhagida and Homalorhagida.

Only a single attempt has been made to, at least partially, understand kinorhynch phylogeny through numerical analysis of morphological characters [18]. However, this study focused on the family Echinoderidae only, and the results were not that conclusive. More recently, two studies have addressed general kinorhynch interrelationships [19], [20]. Both studies were based on analysis of molecular sequence data ([19]: 18S rRNA; [20]: 18S rRNA+28S rRNA) and attempted to cover a broad variety of kinorhynch taxa. The results of the two studies were also congruent in many respects. They partly confirmed some groupings from the traditional classification, but they also pointed out new, more surprising clades that had not been proposed previously. The most significant and interesting difference from the traditional classification was perhaps the apparent polyphyly of Cyclorhagida, due to the cyclorhagid genus *Dracoderes* that in most analyses branched out as sister clade to Homalorhagida, or even as a homalorhagid ingroup.

A downside for both studies though was the taxon sampling that still was incomplete. If the objective is to analyze the phylogenetic relationships within a phylum with only 23 genera, an apparently feasible criterion would be to aim for a taxon sampling with at least one or two representatives for each genus. However, for a group like Kinorhyncha this is not as simple as it may sound. Many species have rather restricted distributional ranges, and since several genera accommodate only one or a few species, known from the deep-sea or very remote localities, it may be challenging to get access to tissue for molecular sequencing. Hence, for now, a taxon sampling covering all genera is not realistic.

In the present contribution we have attempted to improve the taxon sampling issue though. First of all, we are able to present molecular sequence data from the so far most comprehensive ingroup taxon sample. Secondly, we have included information from morphological data. The latter enables us to supplement the molecular data with information from a second data source, but, maybe more importantly, it also allows us to include taxa of genera for which molecular sequence data are not currently available. As a result, we are here able to present the so far most comprehensive analyses of kinorhynch interrelationships, and at the same time, the first kinorhynch phylogeny inferred from a combined approach of molecular loci and morphology.

## Materials and Methods

### Taxon sampling

The ingroup comprises 60 kinorhynch species (Table 1), representing all 23 described genera and one undescribed. Information from all 60 species is included in the morphological data matrix. The molecular data includes information from the loci 18S rRNA and 28S rRNA. The molecular taxon sampling is the result of an effort that was initiated in 2004, and hence has been going on for more than ten years. By that time, 18S rRNA and 28S rRNA were the most commonly used loci for phylogenetic analyses of taxa with deep splits, and even though a different approach would have been used if the study was about to be started over today, the choice of loci should be understood in light of the long-term sampling effort.

**Table 1:** Species included in the analyses. The six outgroup taxa, *Priapulus caudatus* and *Halicryptus spinulosus* (Priapulida), *Chordodes morgani* and *Gordius aquaticus* (Nematomorpha), and *Xiphinema rivesi* and *Trichinella spiralis* (Nematoda), and 61 kinorhynch ingroup taxa, representing 60 kinorhynch species; inclusive collecting localities for specimens used for sequencing, accession numbers for sequences and source for coding of morphological characters.

Genus and species	18S rRNA	28S rRNA	Morphology references	Locality of sequenced specimens
*Antygomonas caeciliae*	KF372857	–	[57]	Meloria Shoals, Italy, Mediterranean
*Antygomonas paulae*	LC007036	–	[58]	Fort Pierce, Florida, Northwest Atlantic
*Antygomonas* sp. 2	AB738340	AB738341	Yamasaki and Sørensen (pers. obs.)	Nagasaki, Japan, Northwest Pacific
*Antygomonas* sp. 3	AB738342	AB738343	Yamasaki and Sørensen (pers. obs.)	Okinawa, Japan, Northwest Pacific
*Campyloderes* cf. *vanhoeffeni*	LC007037	–	[46]	Kaldbak, Faroe Islands, North Atlantic
*Campyloderes* sp. 1	AB738344	AB738345	Yamasaki and Sørensen (pers. obs.)	Okinawa, Japan, Northwest Pacific
*Campyloderes* sp. 2	AB738346	AB738347	Yamasaki and Sørensen (pers. obs.)	Okinawa, Japan, Northwest Pacific
*Cateria gerlachi*	–	–	[59]	–
*Centroderes spinosus*	KF372858	–	[48]	Chioggia, Italy, Mediterranean
*Centroderes* sp.	LC008445	LC008446	Yamasaki and Sørensen (pers. obs.)	Off Amami Island, Japan, Northwest Pacific
*Cephalorhyncha* sp. 1	AB738352	AB738353	Yamasaki and Sørensen (pers. obs.)	Hokkaido, Japan, Northwest Pacific
*Condyloderes* sp. 1	LC007038	LC007062	Sørensen (pers. obs.)	Uljin, Korea, Korean East Sea
*Condyloderes* sp. 2	LC007039	LC007063	Herranz (pers. obs.)	Naples, Italy, Mediterranean
*Dracoderes abei*	AB738350	AB738351	[31, 32]	Seto Inland Sea, Japan
*Dracoderes nidhug*	LC007040	LC007064	[33]	Uljin, Korea, East Sea
*Echinoderes ajax*	LC007041	–	[43]	São Sebastião, Brazil, Southwest Atlantic
*Echinoderes astridae*	LC007042	–	[43]	São Sebastião, Brazil, Southwest Atlantic
*Echinoderes aureus*	LC007043	–	[39]	Shirahama, Wakayama, Japan
*Echinoderes capitatus*	KF372859	–	[60, 61]	Chioggia, Italy, Mediterranean
*Echinoderes dujardinii*	LC007044	LC007065	[62]	Ceuta, Spain, Mediterranean Sea
*Echinoderes gerardi*	KF372860	–	[63]	Castellammare, Italy, Mediterranean
*Echinoderes horni*	EU669453	–	[37]	Fort Pierce, Florida, Northwest Atlantic
*Echinoderes marthae*	LC007045	–	[43]	São Sebastião, Brazil, Southwest Atlantic
*Echinoderes microaperturus*	LC007046	LC007066	[41]	Korea Strait
*Echinoderes sensibilis*	LC007047	–	[64]	Shirahama, Wakayama, Japan
*Echinoderes setiger*	KF372864	–	Dal Zotto (pers. obs.)	Castellammare, Italy, Mediterranean
*Echinoderes spinifurca*	EU669455	–	[65]	Fort Pierce, Florida, Northwest Atlantic
*Echinoderes truncatus*	EU669456	–	[35, 37, 66]	Bocas del Toro, Panama, Northwest Atlantic
*Echinoderes* sp. 1	LC007048	–	Sørensen (pers. obs.)	Uljin, Korea, Korean East Sea
*Fissuroderes sorenseni*	–	–	[42]	–
*Fissuroderes thermoi*	–	–	[34]	–
*Franciscideres kalenesos*	KF372869	–	[19]	Ilhabela, Brazil, Southwest Atlantic
*Kinorhynchus giganteus*	KF372863	–	[17], Sánchez (pers. obs)	Castellammare, Italy, Mediterranean
*Kinorhynchs yushini*	AB738370	AB738371	[17], Sørensen (pers. obs)	Oshoro Bay, Japan, Northwest Pacific
*Meristoderes macracanthus*	LC007049	LC007067	[35]	Naples, Italy, Mediterranean
*Meristoderes* sp. 1	LC007050	LC007068	Sørensen (pers. obs.)	Okinawa, Japan, North West Pacific
*Mixtophyes abyssalis*	–	–	[5]	–
*Neocentrophyes intermedius*	–	–	[67]	–
*Neocentrophyes satyai*	–	–	[67]	–
*New Genus*	AB738378	AB738378	Yamasaki and Sørensen (pers. obs.)	Kagoshima, Japan, Northwest Pacific
*Paracentrophyes anurus*	AB738368	AB738368	[68]	East China Sea
*Paracentrophyes quadridentatus*	LC007051	LC007076–77	[68]	18S: Espegrend, Norway, Northeast Atlantic. 28S: Naples, Italy, Mediterranean Sea
*Polacanthoderes martinezi*	–	–	[69], Sørensen (pers. obs.)	–
*Pycnophyes communis*	KF372867		Sánchez (pers. obs)	Cesenatico, Italy, Mediterranean
*Pycnophyes dentatus*	LC007052	LC007069	Sánchez (pers. obs)	Ceuta, Spain, Mediterranean Sea
*Pycnophyes greenlandicus*	AY428820		[38], Sørensen (pers. obs.)	Disko Island, Greenland, Northwest Atlantic
*Pycnophyes kielensis*	U67997		Sørensen (pers. obs.)	NW Germany, Northeast Atlantic
*Pycnophyes oshoroensis*	AB738372	AB738373	[70]	Hokkaido, Japan, Northwest Pacific
*Pycnophyes robustus*	LC007053	LC007070	Sánchez (pers. obs)	Naples, Italy, Mediterranean
*Pycnophyes tubuliferus*	LC007054	–	[17], Sørensen (pers. obs)	East China Sea
*Pycnophyes zelinkaei*	LC007055	LC007071	Sánchez (pers. obs)	Naples, Italy, Mediterranean Sea
*Semnoderes armiger* (Italy)	LC007056	LC007072	[71], Dal Zotto (pers. obs.)	Naples, Italy, Mediterranean Sea
*Semnoderes armiger* (Norway)	LC007057	LC007073	[71], Sørensen (pers. obs.)	Espegrend, Norway, Northeast Atlantic
*Sphenoderes poseidon*	AB738364	AB738365	[72]	Okinawa, Japan, Northwest Pacific
*Triodontoderes anulap*	LC007058	LC007074	[73]	Micronesia, Southwest Pacific
*Tubulideres seminoli*	LC007059	LC007075	[74]	Fort Pierce, Florida, Northwest Atlantic
*Wollunquaderes majkenae*	LC007060	–	[75]	Coral Sea, NE Australia, Southwest Pacific
*Zelinkaderes brightae*	LC007061	–	[74]	Fort Pierce, Florida, Northwest Atlantic
*Zelinkaderes klepali*	KF372868	–	[76]	Marsa Mubarak, Egypt, Red Sea
*Zelinkaderes yong*	AY746985	–	[77]	Guryongpo, Korea, Korean East Sea
*Zelinkaderes* sp. 1	AB738366	AB738367	Yamasaki and Sørensen (pers. obs.)	Kagoshima, Japan, Northwest Pacific
*Priapulus caudatus* (outgroup)	X87984	AY210840	–	?
*Halicryptus spinulosus* (outgroup)	AF342790	AF342789	–	?
*Chordodes morgani* (outgroup)	AF036639	AF342787	–	?
*Gordius aquaticus* (outgroup)	X80233	AY210817	–	?
*Xiphinema rivesi* (outgroup)	HM921344	Ay210845	–	?
*Trichinella spiralis* (outgroup)	U60231	AF342803	–	?

The molecular sequence data includes 54 sequences of 18S rRNA. The data represents 53 distinct species (one of them, *Semnoderes armiger*, is represented by two populations with slightly different sequences), of which one is a new, yet undescribed genus (= New Genus in the following) and further ten are either new species, or species that could not be identified with certainty. In total, the 53 species represents 18 genera. The five genera *Cateria*, *Fissuroderes*, *Mixtophyes*, *Neocentrophyes* and *Polacanthoderes* are hence only represented by morphology. The 28S rRNA fragment did not amplify easily for all species, and sequences were only obtained for 24 species. Outgroup taxa for the molecular analyses includes the priapulids *Priapulus caudatus* and *Halicryptus spinulosus*, the nematomorphs *Chordodes morgani* and *Gordius aquaticus*, and the nematodes *Xiphinema rivesi* and *Trichinella spiralis*.

### Molecular sequencing

DNA extraction, amplification and sequencing were carried out by several of the authors that used slightly different approaches. All relevant information regarding DNA extraction and acquisition of sequences is summarized in Table 2.

**Table 2:** Extraction, primer, PCR setting and sequencing information. Summary of the choice of DNA extraction kit, primers, PCR settings and sequencing information, used by the four authors, HSR, HY, MDZ and MVS. 28S rRNA primers used by HSR are new and have not been published previously.

HSR	Extraction	QIAamp tissue kit
	Primers 18S	328 [78]: 5’ TACCTGGTTGATCCTGCCAG 3’
		R [78]: 5’ AAAGATTAAGCCATGCATGT 3’
		A- [78]: 5’ TGGAGGGCAAGTCTGGTG 3’
		A [78]: 5’ CTGGCACCAGACTTGCCCT 3’
		G- [78]: 5’ AGAGGTGAAATTCTTGGA 3’
		G [78]: 5’ TCCAAGAATTTCACCTCT 3’
		I- [78]: 5’ AAACTTAAAGGAATTGACGGA 3’
		I [78]: 5’ TCCGTCAATTCCTTTAAGTTT 3’
		D- [78]: 5’ TGTGATGCCCTTAGA 3’
		D [78]: 5’ TCTAAGGGCATCACA 3’
		T [78]: 5’ ACCTTGTTACGACTTTTA 3'
		329 [78]: 5’ TAATGATCCTTCCGCAGGTT 3’
	Primers 28S	NLF184-1: 5’ GGAGGAAAAGAAACTAAC 3’
		NLR512: 5’ TACTTGTYBRCTATCG 3’
		NLF796: 5’ GTCTTGAAACACGGACCAAGG 3’
		NLF1410: 5’ TCCGCTAAGGAGTGTGTAACAAC 3’
		NLR1432: 5’ GTTGTTACACACTCCTTAGCGGA 3’
		NLR2098: 5’ AGCCAATCCTTWTCCCGAAGTTAC 3’
		NLR3113: 5’ GTCTAAACCCAGCTCACGTTCCCT 3’
	PCR settings	3 min at 94°C-> 30 cycles: (1 min at 94°C-> 1 min at 54.5°C-> 2 min at 72°C)-> 10 min at 72°C
	Purification	GeneClean (Bio 101) Nal/glass-powder Kit
	Sequencing	ABI PRISM 3100 automated DNA Analyzer
HY	Extraction	QIAGEN DNeasy Tissue Kit
	Primers 18S	F1 [79]: 5’ TACCTGGTTGATCCTGCCAG 3’
		F2 [79]: 5’ CCTGAGAAACGGCTRCCACAT 3’
		F3 [79]: 5’ GYGRTCAGATACCRCCSTAGTT 3’
		F4 [79]: 5’ GGTCTGTGATGCCCTYAGATGT 3’
		R6 [79]: 5’ TYTCTCRKGCTBCCTCTCC 3’
		F7 [79]: 5’ GYYARAACTAGGGCGGTATCTG 3’
		F8 [79]: 5’ ACATCTRAGGGCATCACAGACC 3’
		R9 [79]: 5’ GATCCTTCCGCAGGTTCACCTAC 3’
	Primers 28S	28S-01 [80]: 5’ GACTACCCCCTGAATTTAAGCAT 3’
		28S-n05R [20]: 5’ CTCACGGTACTTGTTCGCTAT 3’
		28SR-01 [80]: 5’ GACTCCTTGGTCCGTGTTTCAAG 3’
		28Sf [81]: 5’ TGGGACCCGAAAGATGGTG 3’
		28S-15R [20]: 5’ CGATTAGTCTTTCGCCCCTA 3’
		28Sr [81]: 5’ ACACACTCCTTAGCGGA 3’
		28S-2KF [20]: 5’ TTGGAATCCGCTAAGGAGTG 3’
		28S-3KF [20]: 5’ AGGTGAACAGCCTCTAGTCG 3’
		28S-3KR [20]: 5’ CCAATCCTTTTCCCGAAGTT 3’
		28v-5’ [82]: 5’ AAGGTAGCCAAATGCCTCATC 3’
		28S-42F [20]: 5’ GAGTTTGACTGGGGCGGTA 3’
		28jj-3’ [82]: 5’ AGTAGGGTAAAACTAACCT 3’
	PCR settings	1 min at 95°C-> 35 cycles: (30 sec at 95°C-> 90 sec at 45°C-> 3 min at 72°C)-> 7 min at 72°C
	Purification	Exo-Sap
	Sequencing	Life Technologies 3730 DNA Analyzer
MDZ	Extraction	QIAGEN QIAmp Micro Kit
	Primers 18S	S30 [83]: 5’ GCTTGTCTCAAAGATTAAGCC 3’
		5FK [84]: 5’ TTCTTGGCAAATGCTTTCGC 3’
		4FB [85]: 5’ CCAGCAGCCGCGGTAATTCCAG 3’
		1806R [83]: 5’ CCTTGTTACGACTTTTACTTCCTC 3’
	PCR settings	3 min at 95°C-> 40 cycles: (30 sec at 94°C-> 30 sec at 52°C-> 30 sec at 72°C)-> 10 min at 72°C
	Purification	NucleoSpin Extra Kit
	Sequencing	Macrogen Inc.
MVS	Extraction	QIAGEN DNeasy Tissue Kit
	Primers 18S	1F [86]: 5’ TACCTGGTTGATCCTGCCAGTAG 3’
		4R [86]: 5’ GAATTACCGCGGCTGCTGG 3’
		3F [86]: 5’ GTTCGATTCCGGAGAGGGA 3’
		18S bi [87]: 5’ GAGTCTCGTTCGTTATCGGA 3’
		18S a2.0 [87]: 5’ ATGGTTGCAAAGCTGAAAC 3’
		9R [86]: 5’ GATCCTTCCGCAGGTTCACCTAC 3’
	Primers 28S	28S D1F [88]: 5’ GGGACTACCCCCTGAATTTAAGCAT 3’
		28S b [87]: 5’ TCGGAAGGAACCAGCTACTA 3’
	PCR settings	1 min at 94°C-> 40 cycles: (30 sec at 94°C-> 30 sec at 47°C-> 1 min at 72°C)-> 6 min at 72°C
	Purification	QIAquick PCR Purification Kit
	Sequencing	ABI 3730 genetic analyzer

### The morphological matrix

The morphological matrix includes 42 informative characters. 32 are binary and ten are multistate characters, representing a total of 102 characters states. Seven multistate characters were treated as unordered whereas three were treated as ordered, with symmetrical character transformations. The morphological characters are listed in Appendix A: The morphological characters (**S1 File**), and the morphological character matrix is presented in Appendix B: The morphological matrix (**S2 File**). The data matrix was compiled in NEXUS Data Editor ver. 0.5.0 [21].

The morphological dataset does not include any outgroup taxa. This was the consequence after several attempts to include priapulid, nematode, nematomorphs, or even loriciferan, outgroup taxa. It turned out, however, that even though a close relationship, at least with priapulids, hardly can be questioned, only extremely few ingroup resolving character traits were applicable for the potential outgroup taxa.

### Data analyses

The data were analyzed in different combinations: Morphological data were analyzed alone, and 18S rRNA, and 28S rRNA data were analyzed separately and in combination. Furthermore, analyses of 28S rRNA alone and combined 18S rRNA + 28S rRNA were carried out on a restricted dataset where sequences of *Tubulideres seminoli* and *Pycnophyes robustus* were omitted because the 28S rRNA fragments of these two species contained much missing data compared to the other taxa. Finally, all data (18S rRNA- 28S rRNA—morphology) were combined and analyzed.

Maximum parsimony (MP) analysis of the morphological data was processed with TNT ver. 1.1 [22], using New Technology Search. Equal weights and otherwise default settings were assigned to all characters, besides the characters 16, 17 and 19 that were treated as additive.

Sequences from each gene were pre-aligned separately with MAFFT software [23], [24] using the FFT-NS-2 option and were subsequently divided into domains by eye. Domain sequences were realigned individually with MAFFT software using the L-INS-i option. Alignment-ambiguous positions were removed with TrimAl software [25] in “strict setting”, and all positions bearing gaps were also removed. The trimmed domain sequences were recombined to form the final dataset for the analyses. Homogeneities of base frequencies and optional substitution models for 18S rRNA alone, 28S rRNA alone, and 18S rRNA + 28S rRNA datasets were tested with Kakusan4 [26]. The homogeneity test indicated that the base composition of each dataset was significantly homogeneous. Maximum likelihood (ML) trees of all molecular datasets (18S rRNA, 28S rRNA, and 18S rRNA + 28S rRNA datasets) were constructed with raxmlGUI 1.2 [27], [28]. Bayesian inference (BI) trees of all molecular and morphological + molecular datasets were constructed with MrBayes 3.2.1 [29]. MP analysis of morphological + molecular dataset was also done with the same software and settings as the morphological analysis.

Morphological character optimizations in trees combining molecular and morphological data were explored in Mesquite Ver. 3.01 [30].

## Results

### The morphological data analysis

**Fig 1:**
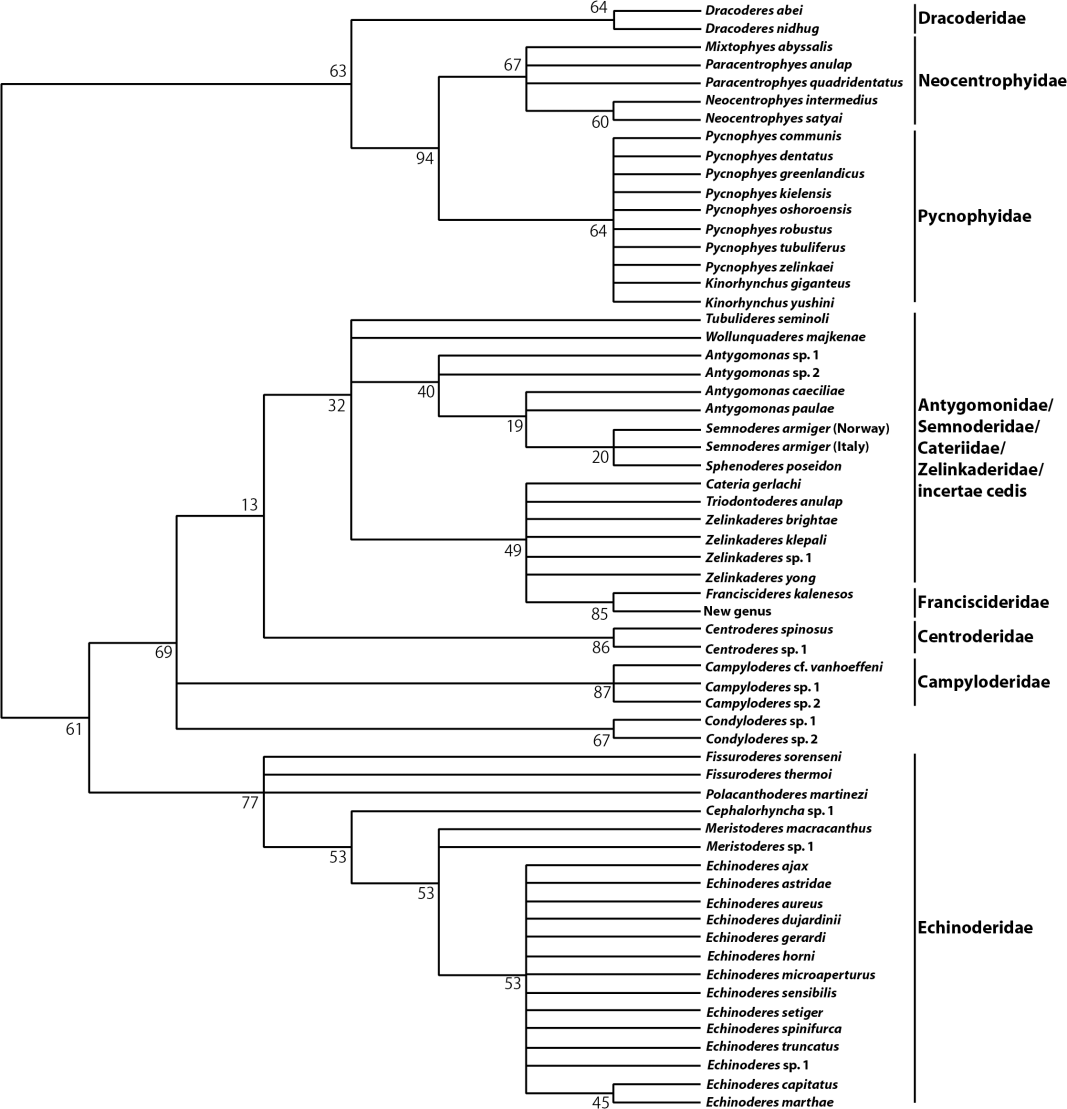
Morphological tree. Unrooted strict consensus of five most parsimonious trees of score 83, obtained from New Technology Search in TNT. Numbers at branches indicate bootstrap values.

The New Technology Search of data from the morphological matrix resulted in five most parsimonious trees (Tree length: 82). A strict consensus tree, based on the five most parsimonious trees is shown in Fig 1. Since the analysis, as stated above, was carried out without outgroup taxa, the tree should be seen as unrooted. However, in the strict consensus tree, we chose to set a root that would correspond to the rooting of the tree obtained by BI of the combined morphological and molecular datasets. We find that this can be justified, since the two trees are based on the same morphological information, and that the molecular data in this context can be seen as supplementary information that enables a better outgroup comparison and rooting of the tree.

The five most parsimonious trees all show the same basal topologies, but the topologies are very different in the distal parts of the trees. Common for all trees is a basal division into two major clades. One clade consists of two species of *Dracoderes* that appears as sister group to a clade accommodating species of the traditional homalorhagid genera, i.e., *Pycnophyes*, *Kinorhynchus*, *Mixtophyes*, *Paracentrophyes*, and *Neocentrophyes*. The three latter always form a monophyletic group, whereas species of *Pycnophyes* and *Kinorhynchus* form a second clade together. Within these two clades, the relationships remain unresolved.

The other major clade corresponds to the traditional cyclorhagid order, but without *Dracoderes*. Also this clade splits into two groups. One group accommodates all echinoderid taxa, whereas the other contains all taxa with a midterminal spine. Within the echinoderid clade, monophyly of *Echinoderes* is supported, whereas the relationships between the remaining echinoderid taxa remain unresolved. In the other major clade, characterized by species with midterminal spine, species of *Campyloderes* and *Condyloderes* always branch off as the first two groups, followed by *Centroderes*. Each of the three genera always appears monophyletic, but they never form a monophyletic group together. The relationships between the remaining taxa, that form a large sister clade to *Centroderes*, are completely unresolved. The only consistent trends are a close relationship between *Franciscideres* and New Genus, and monophyly of the semnoderid taxa, *Semnoderes* and *Sphenoderes*.

### The molecular data analyses

**Fig 2:**
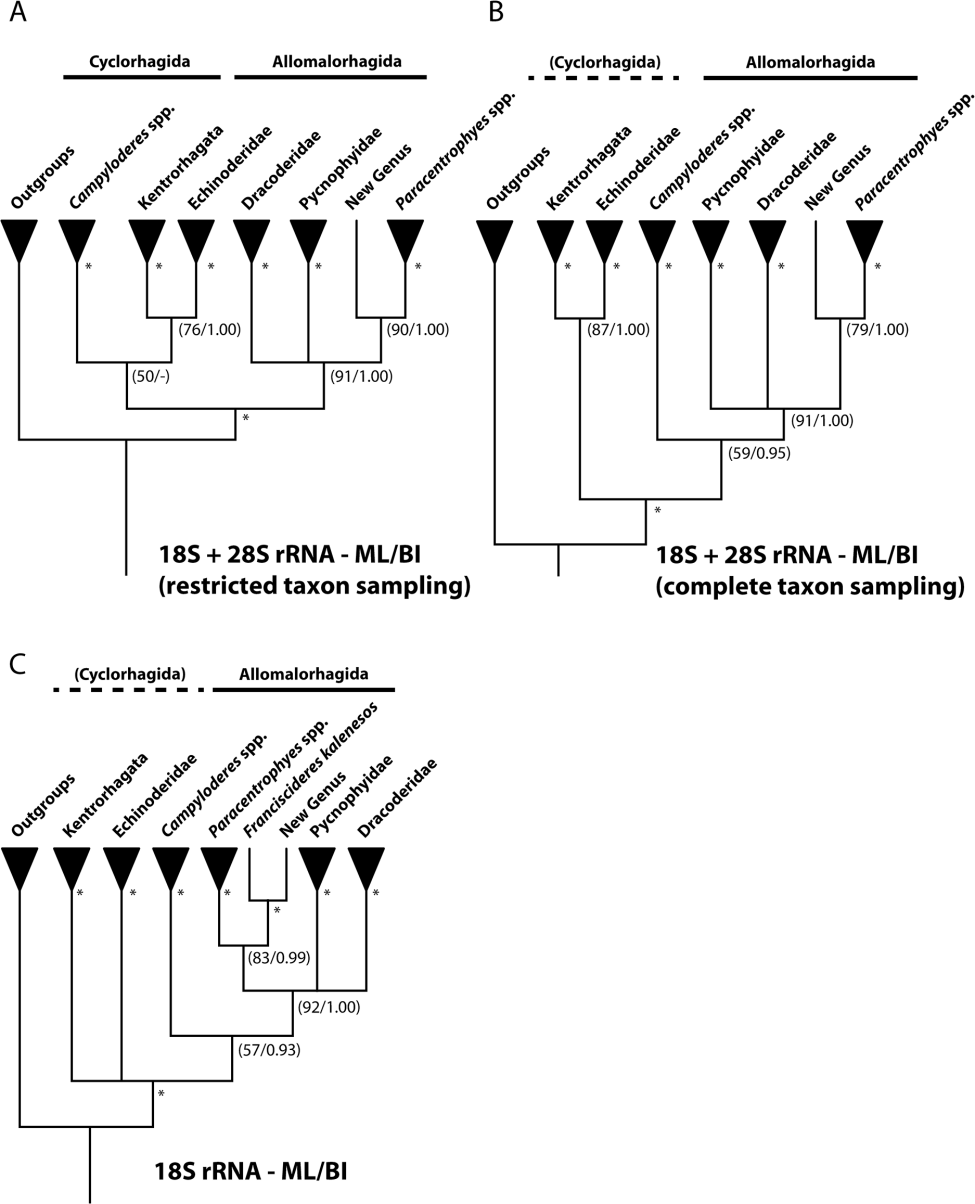
Summary trees showing results from analyses of the molecular sequence data. A. Maximum Likelihood analysis and Bayesian Inference of combined 18S rRNA and 28S rRNA. Taxa with much missing information in the 28S rRNA marker have been omitted from these analyses. B. Maximum Likelihood analysis and Bayesian Inference of combined 18S rRNA and 28S rRNA. All taxa, including those with much missing information in the 28S rRNA marker, are included in these analyses. C. Maximum Likelihood analysis and Bayesian Inference of 18S rRNA. Support measures are indicated at the internodes as: ML bootstrap value/BI posterior probability. * means bootstrap value>95 + posterior probability = 1.00. Clades with ML bootstrap values <50 and BI posterior probabilities <0.95 are collapsed.

The resulting trees from the different analyses of molecular sequence data are presented in Fig 2: ML and BI of combined 18S rRNA and 28S rRNA sequences from the restricted taxon sampling (Fig 2A), ML and BI of all 18S rRNA and 28S rRNA sequences combined (Fig 2B), and ML and BI of 18S rRNA (Fig 2C). All analyses basically produce the same major clades, but the rooting of the resulting trees and the interrelationships between the major clades differs greatly (Fig 2). Following clades are supported in all analyses: *Campyloderes*, Echinoderidae, Dracoderidae, Pycnophyidae, *Paracentrophyes*, a large clade accommodating all taxa (except *Campyloderes*) with midterminal spine (named Kentrorhagata in Fig 2), and a clade consisting of *Paracentrophyes*, New Genus, and *Franciscideres* (with the latter only included in the 18S rRNA analyses). All trees furthermore support monophyly of a clade consisting of Dracoderidae, Pycnophyidae, *Paracentrophyes*, and New Genus (plus *Franciscideres* when included). This clade is indicated as Allomalorhagida in Fig 2. Within the different major clades the results are much more incongruent, and in general it appears that the data cannot clarify the relationships in the distal parts of the trees consistently. Since the results from the distal parts of the trees obviously can be disregarded, we have, for simplicity, chosen to present the trees with taxa of different monophyletic groups merged into single branches. Generally ML and BI analyses of each dataset produced very similar trees that only differed in regard to the relationships within the merged groups. The resulting trees from analyses of 28S rRNA all supported the major clades mentioned here, but otherwise they left the relationships between these clades completely unresolved, hence, these trees will not be discussed any further.

The clade indicated as ‘Allomalorhagida’ includes taxa of *Pycnophyes*, *Kinorhynchus*, and *Paracentrophyes*, i.e., classic homalorhagid taxa, together with *Dracoderes*, the yet undescribed genus, and *Franciscideres*. The three remaining clades, *Campyloderes*, ‘Kentrorhagata’ and Echinoderidae form a monophyletic group in trees based on the restricted dataset with combined 18S rRNA and 28S rRNA (Fig 2A), and besides Dracoderidae, that appear within ‘Allomalorhagida’, this clade corresponds to the traditional group Cyclorhagida. In the remaining trees (Fig 2B and 2C), Cyclorhagida is not supported because *Campyloderes* appears as sister group to ‘Allomalorhagida’. Echinoderidae and ‘Kentrorhagata’ always appear as either sister clades, or in a trichotomy together with a clade accommodating all other kinorhynchs.

### The combined molecular and morphological data analyses

The MP analysis supported two clades with homalorhagid taxa, i.e., *Mixtophyes* grouping with *Paracentrophyes* and *Neocentrophyes*, and species of *Pycnophyes* mixed with *Kinorhynchus*. It also supported monophyletic Echinoderidae, *Campyloderes*, a clade with *Franciscideres* plus New Genus, and a clade with taxa of *Condyloderes*, *Centroderes*, *Wollunquaderes*, *Tubulideres*, *Triodontoderes*, *Zelinkaderes*, *Sphenoderes*, *Semnoderes*, *Antygomonas*, and *Cateria*. However, besides these clades, the analysis failed to provide much resolution, and all clades were left in polytomy. Due to this lack of resolution, we chose to disregard this tree in the following, and focus on the results of the BI analysis.

**Fig 3:**
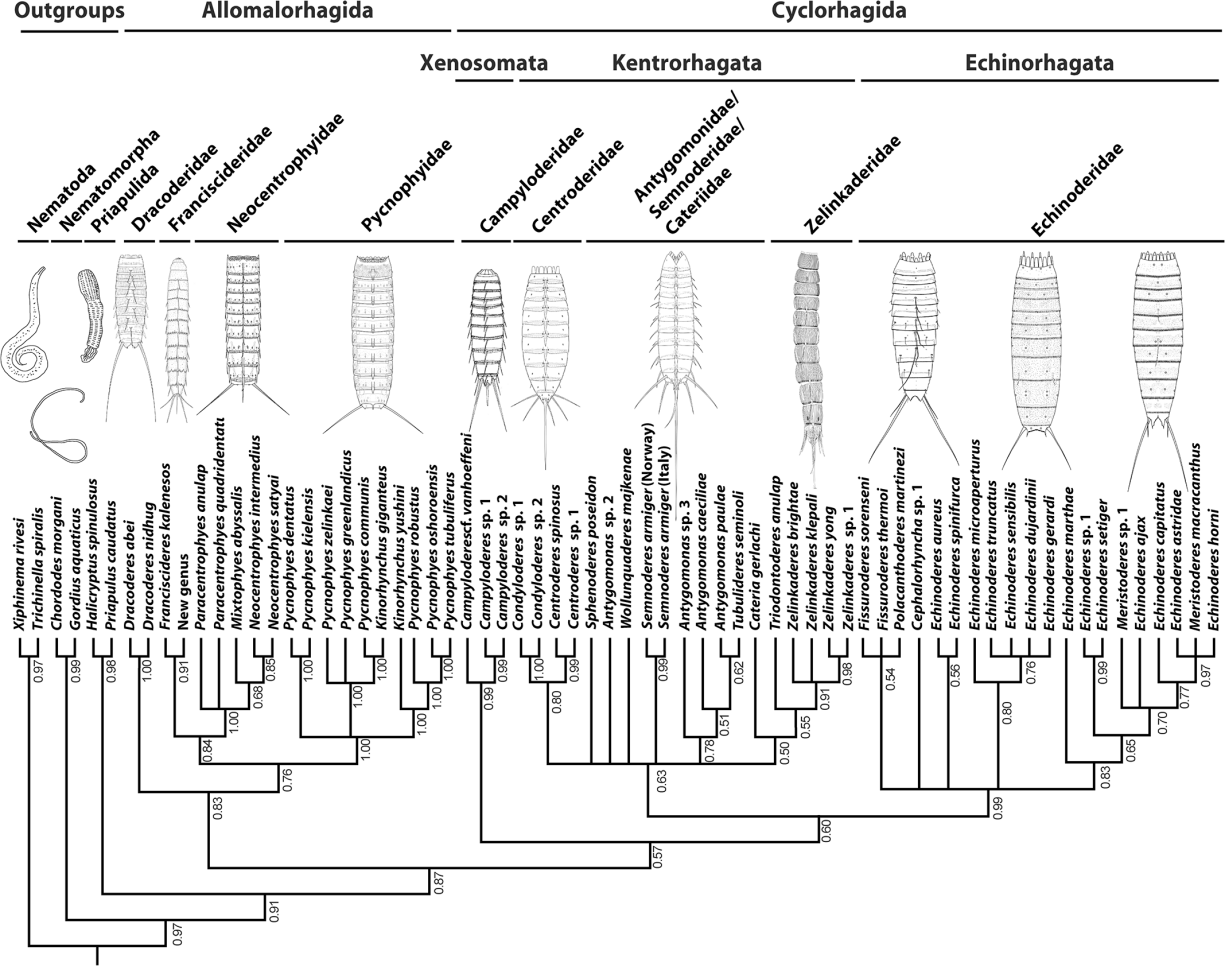
Combined morphological and molecular tree. Tree resulting from Bayesian Inference of combined morphological and molecular data sets. Numbers at branches indicate posterior probabilities.

BI analysis of combined molecular and morphological data produced a tree that can be divided into two major clades (Fig 3). One major clade includes taxa of *Dracoderes*, *Franciscideres*, New Genus, *Mixtophyes*, *Paracentrophyes*, *Neocentrophyes*, *Pycnophyes* and *Kinorhynchus*. The *Dracoderes* species branch off as the most basal taxa of the clade. The remaining taxa form two clades, one with species of *Pycnophyes* and *Kinorhynchus* mixed together, and one with New Genus and *Franciscideres* appearing as sister taxa, and together forming the sister group to a clade with *Mixtophyes*, *Paracentrophyes* and *Neocentrophyes*.

The other major kinorhynch clade includes all cyclorhagids, except species of *Dracoderes*. Three major clades appear within this group: Echinoderidae, *Campyloderes* and a clade with all remaining taxa with midterminal spine (in Fig 3 marked as Echinorhagata, Xenosomata and Kentrorhagata). *Campyloderes* appears as the most basal clade, whereas Echinoderidae forms the sister clade to the group including all remaining taxa with midterminal spine. Inside Echinoderidae we see some resolution, but the validity of the topology is questionable. Taxa of *Fissuroderes* and *Polacanthoderes* are united in a trichotomy (this clade is supported by morphological data only), whereas taxa of *Echinoderes*, *Cephalorhyncha* and *Meristoderes* are mixed together. There is no indication of monophyly for *Echinoderes* or *Meristoderes*, and *Meristoderes* and *Cephalorhyncha* are not grouped together.

In the third cyclorhagid clade, accommodating exclusively species with midterminal spine, monophyly is supported at the generic level for *Condyloderes*, *Centroderes*, *Semnoderes*, and *Zelinkaderes* but not for *Antygomonas*. Furthemore, the family Zelinkaderidae, which includes *Zelinkaderes* and *Triodontoderes*, appears as monophyletic, and *Condyloderes* and *Centroderes* as sister groups. Otherwise, species of *Wollunquaderes*, *Tubulideres*, *Sphenoderes*, *Semnoderes*, *Antygomonas* and *Cateria* appear to be mixed randomly together, and we do not consider these topologies as valid.

## Discussion

### Evaluation of the obtained tree topologies

All obtained trees obviously present a mix of congruent results, and results that appear to be somehow more random. Roughly, it appears that the major groupings show a high degree of congruence across the different data and analytical approaches, whereas the topologies in the distal parts of the trees are much more questionable. As we see it, this is a result of a clear limitation of the available molecular information that apparently is unable to resolve relationships between more closely related taxa.

#### The basal splits, and position of *Franciscideres*, New Genus and *Cateria*

Despite the lack of resolution in the distal parts of the tree, the congruent results allow us to identify some major clades that we consider well-supported and valid. Most interestingly, we see that most analyses with information from all molecular markers data (Figs 2A and 3), divide Kinorhyncha into two major clades: one with the traditional homalorhagid taxa together with *Dracoderes*, New Genus and *Franciscideres*, and one that, besides *Dracoderes*, accommodates all cyclorhagid taxa. This basal division also corresponds well with the results from the morphological analysis, but with one difference, namely the position of New Genus and *Franciscideres*, that according to morphology should be grouped with the cyclorhagids. The morphological analysis (Fig 1) places New Genus and *Franciscideres* in an otherwise unresolved clade, together with *Cateria*, *Triodontoderes* and *Zelinkaderes*. From a morphological point of view, a clade like this is not unlikely, and as discussed by Dal Zotto et al. [19] these particular taxa show some clear affinities. According to Dal Zotto et al. [19], especially *Cateria* appears to be a very likely sister clade to the New Genus and *Franciscideres*, but since molecular sequence data is still unavailable from *Cateria*, we cannot test this hypothesis. Hence, until such data become available for *Cateria*, we chose to follow the results of the combined molecular and morphological analysis, and consider New Genus and *Franciscideres* as more closely related with the traditional homalorhagid taxa, whereas *Cateria* for now remains with the cyclorhagids.

#### A new basal kinorhynch clade, and the end of homalorhagid monophyly

The major clade consisting of *Dracoderes*, New Genus and *Franciscideres*, and the homalorhagid taxa is supported by ML and BI analyses of 18S rRNA and combined 18S rRNA and 28S rRNA (Fig 2A, 2B and 2C), and BI of combined molecular and morphological data (Fig 3). A similar clade was obtained by the recent molecular analyses of Yamasaki et al. [20] and Dal Zotto et al. [19], but with the differences that [20] did not include information from *Franciscideres* in their analyses, and [19] did not find support for inclusion of *Paracentrophyes* in the clade. When the results of the present analyses congruently support the inclusion of *Paracentrophyes* in this clade, it might have to do with the improved taxon sampling, and the fact the genus here is represented by two species instead of a single one.

BI of combined molecular and morphological data (Fig 3) supports a monophyletic group consisting of *Franciscideres*, New Genus, *Paracentrophyes*, *Mixtophyes*, and *Neocentrophyes*. The sister-group relationship between, on one hand, *Franciscideres* and New Genus and, on the other, *Paracentrophyes*, *Mixtophyes*, and *Neocentrophyes* may be surprising from morphological perspectives, but we cannot ignore that the molecular analyses clearly support affinities between *Paracentrophyes*, New Genus, and *Franciscideres* (Fig 2) (only in analyses of 18S rRNA for the latter, since information from 28S rRNA was not available for this species). Besides this slightly surprising relationship, the two clades themselves make good sense. The close relationship between *Franciscideres* and New Genus has already been demonstrated [19], and seems to be supported by morphology (see below). Also the other clade, representing Neocentrophyidae, has previously been discussed as a potentially monophyletic group [5], even though the authors at this time concluded that inclusion of *Mixtophyes* into Neocentrophyidae would produce less parsimonious trees than other alternative scenarios.

The tree showing results of BI of combined molecular and morphological data (Fig 3) suggests that the sister clade to Neocentrophyidae + *Franciscideres* and New Genus is constituted by species of *Pycnophyes* and *Kinorhynchus*, i.e., what is usually referred to as Pycnophyidae. However, the purely molecular analyses (Fig 2) did not support this, but left Dracoderidae, Pycnophyidae and Neocentrophyidae + *Franciscideres* and New Genus in a trichotomy. Regarding Pycnophyidae it is noteworthy that no analysis finds support for neither monophyletic *Pycnophyes* nor *Kinorhynchus*. This lack of generic monophyly could be due to insufficient molecular sampling, and perhaps also the result of a bias in the high number of *Pycnophyes* species opposed to only two species of *Kinorhynchus*. However, ongoing studies of Sánchez and collaborators actually indicate that species of the two genera mix together, and that a revision of Pycnophyidae is highly needed.

**Fig 4:**
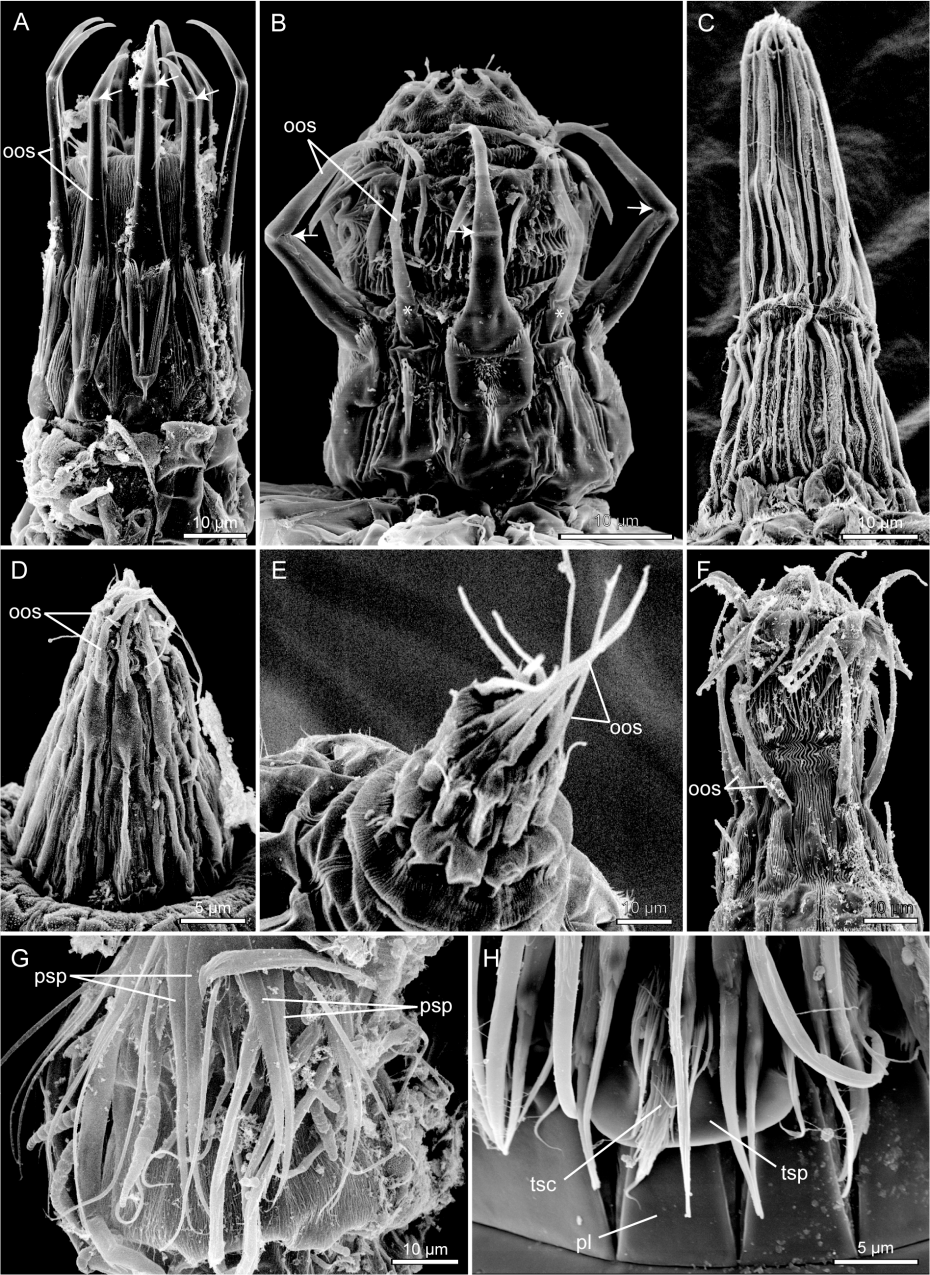
Scanning electron micrographs showing selected morphological character states for characters 1 to 6. A. Mouth cone of *Wollunquaderes majkenae* showing articulated outer oral styles of equal sizes (character 1, state 0; character 2, state 0). B. Mouth cone of *Dracoderes abei*, with articulated outer oral styles alternating in size between larger and smaller (marked with *) ones (character 1, state 1; character 2, state 0). C. Outer oral styles in *Campyloderes* cf. *vanhöffeni*, being either absent or completely fused with mouth cone (character 3, state 2). D. Mouth cone with partly fused outer oral styles of *Condyloderes* sp. 1 (character 3, state 1). E. Mouth cone with soft, non-articulated outer oral styles of *Pycnophyes communis* (character 2, state 1). F. Mouth cone with soft, non-articulated outer oral styles of *Pycnophyes kielensis* (character 2, state 1). G. Introvert of New Genus, showing basally bifurcated primary spinoscalids (character 4, state 1). H. Detail from neck of *Echinoderes microaperturus*, showing a trichoscalid attaching to its trichoscalid plate (character 6, state 1). Abbreviations: oos, outer oral styles; pl, placid; psp, primary spinoscalids; tsc, trichoscalid; tsp, trichoscalid plate. Arrows indicate articulations of outer oral style units.

**Fig 5:**
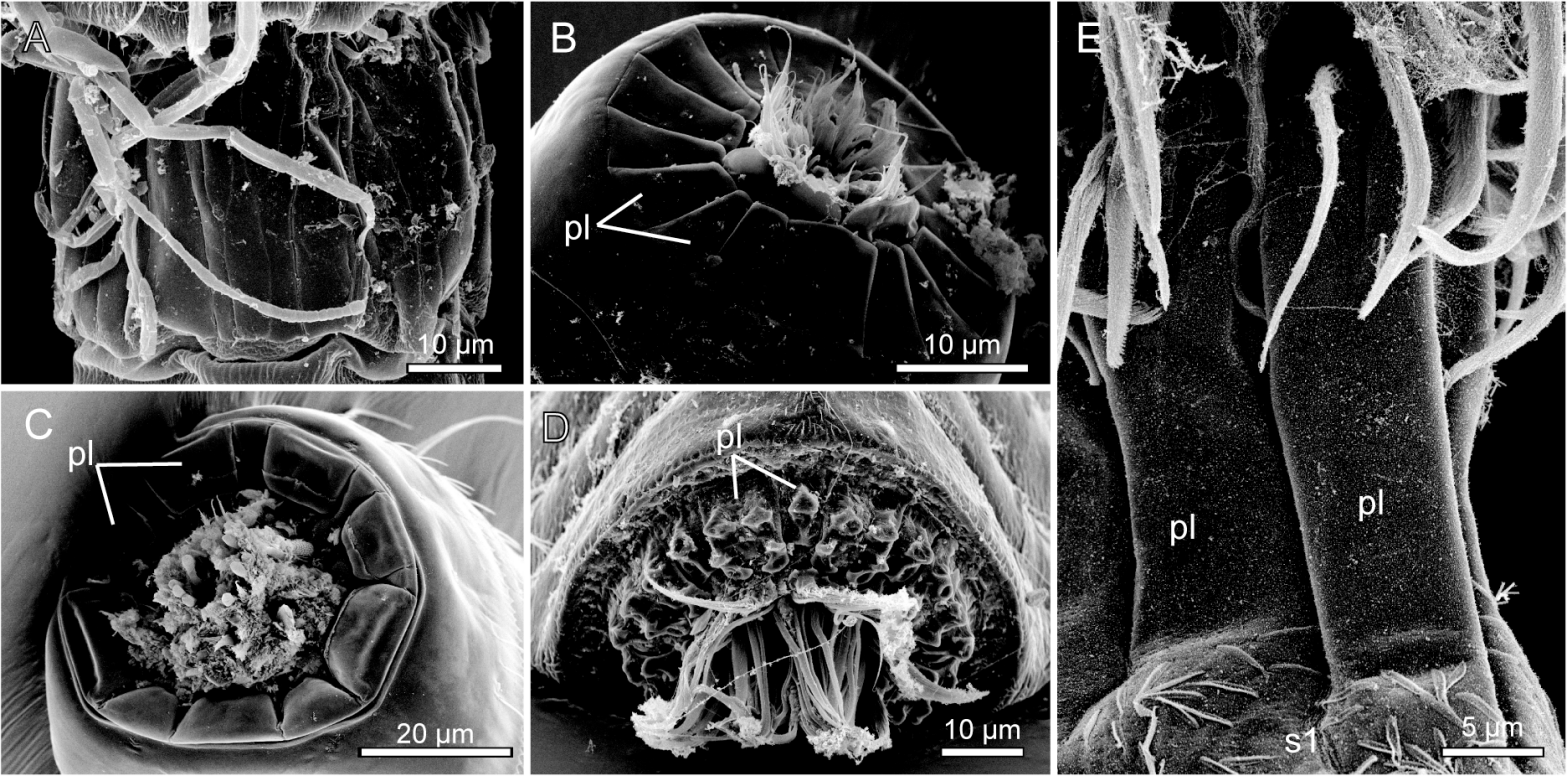
Scanning electron micrographs showing selected morphological character states for characters 7 to 13. A. Segment-like neck region of New Genus (character 7, state 1). B. *Meristoderes macracanthus* with its head retracted into the trunk, showing the closing mechanism with sixteen placids (character 8, state 1; character 9, state 5) arranged in a radial symmetrical pattern (character 13, state 0). Note also the smooth surface of the placids (character 10, state 0), and the distinct articulations between the placids and segment 1 (character 11, state 1). C. *Dracoderes abei* with its head retracted into the trunk, showing the closing mechanism with nine placids (character 8, state 1; character 9, state 3). The spacing between the dorsal placids makes the arrangement of the placids bilateral symmetrical (character 13, state 1). D. *Condyloderes* sp. 1 showing placids with various knobby projections (character 10, state 1). E. Two placids of *Triodontoderes anulap*. Note the distal tripartition (character 11, state 1) and the absence of distinct articulations between the placids and segment 1 (s1) (character 12, state 0). Abbreviations: pl, placids.

MP of the morphological dataset (Fig 1) as well as BI of combined molecular and morphological data (Fig 3) support *Dracoderes* as the most basal clade within this new major kinorhynch clade. *Dracoderes* was originally described as a cyclorhagid genus [31], but recent analyses based on molecular sequence data have indicated that *Dracoderes* is more closely related with the homalorhagid taxa [19], [20]. This option has also been discussed in studies with a much more morphological approach [32], [33], and even though species of *Dracoderes* superficially resemble cyclorhagids, it has been pointed out that species of the genus also share several similarities with the traditional homalorhagid species. These similarities include the conspicuously alternating sizes of the outer oral styles (Fig 4B), the dorsal trichoscalid number and arrangement, and the appearance of the placids in the neck region (Fig 5C) [32], [33]. Hence, considering the congruent results of the present analyses, together with the growing amount of evidence that have appeared in the more recent studies, we find it justified to no longer consider *Dracoderes* as part of the Cyclorhagida. Instead we have clear indications that the genus belongs to this other new kinorhynch assemblage, probably as the most basal taxon as suggested by morphology and analysis of combined morphological and molecular data (Figs 1 and 3).

In conclusion, we find good support for a clade consisting of *Dracoderes*, New Genus, *Franciscideres*, and the homalorhagid taxa. For this clade we propose the name Allomalorhagida nom. nov. (From Greek *Allos*, other, + Homalorhagida). It should be noted that even though we find the traditional homalorhagid taxa inside this clade, we only find support for monophyletic Homalorhagida in the morphological analysis. When molecular sequence data is included, Homalorhagida appears as paraphyletic or polyphyletic This leaves Homalorhagida as a phylogenetically questionable group, and we would recommend that the name is no longer used in a taxonomic context.

#### The phylogeny of the Cyclorhagida

The other major kinorhynch clade that appears in basically all analyses includes all the cyclorhagid taxa, except *Dracoderes* that from this point no longer will be considered a cyclorhagid genus. Within this major clade, all analyses that include molecular sequence data (Figs 2 and 3) support the occurrence of three clades: one with all echinoderid taxa, one with species of *Campyloderes*, and one with all other cyclorhagid taxa. The morphological analysis (Fig 1) recognizes almost the same clades, although *Campyloderes* here occurs in a trichotomy together with *Condyloderes* and a large clade including all other cyclorhagids with midterminal spines. Hence, we find it well-supported that Cyclorhagida can be subdivided into these three clades.

#### The Echinoderidae

One of these three clades accommodates all echinoderid taxa. The clade appears in all analyses, morphological as well as molecular, and support measures are high (>95 bootstrap support and 1.00 posterior probability in all molecular analyses (Fig 2), and 0.99 posterior probability in analysis of combined molecular and morphological data (Fig 3). Hence, we find it clear that monophyly of Echinoderidae, with the present taxon representation, cannot be questioned. Within the clade, we have very little resolution though. The morphological analysis (Fig 1) places the species of *Fissuroderes* and *Polacanthoderes* in a polytomy together with a clade that includes all remaining echinoderids. This result is to some extent a repetition of the result from a previous morphological analysis [18]. Here, the analysis also failed to resolve the relationships between *Fissuroderes* and *Polacanthoderes*. Inside the clade with the remaining echinoderids, the morphological analysis supports that *Cephalorhyncha* branches off first, and appears as sister clade to a trichotomy with the two *Meristoderes* species and monophyletic *Echinoderes*. This topology provides partial support for the idea about *Cephalorhyncha* and *Meristoderes* representing transitional stages between species with segment 2 completely differentiated into a tergal and two sternal plates (i.e. *Fissuroderes* and *Polacanthoderes*) and species with segment 2 forming a closed ring (i.e. *Echinoderes*). This idea has previously been proposed in several studies [3], [18], [34], [35], even though the authors have disagreed about the polarity of this character transformation. However, any support for this hypothesis vanishes in the molecular analysis. The position of *Fissuroderes* and *Polacanthoderes* are obviously only influenced by the signal from the morphological data because molecular sequence data were unavailable for these taxa, but it is noteworthy that the introduction of molecular data for species of *Meristoderes* and *Cephalorhyncha* affects that they mix together with the numerous species of *Echinoderes*, and that not even *Meristoderes* monophyly can be confirmed. As noted above, our dataset has clear limitations, and the topologies in the distal parts of the trees can obviously be questioned. However, the result may still indicate that we have to rethink our ideas about echinoderid phylogeny in future studies. Perhaps too much emphasis has been put on the composition of segment 2, while other phylogenetically important characters have been neglected in the phylogenetic scenario of Echinoderidae supposed up to now.

**Fig 6:**
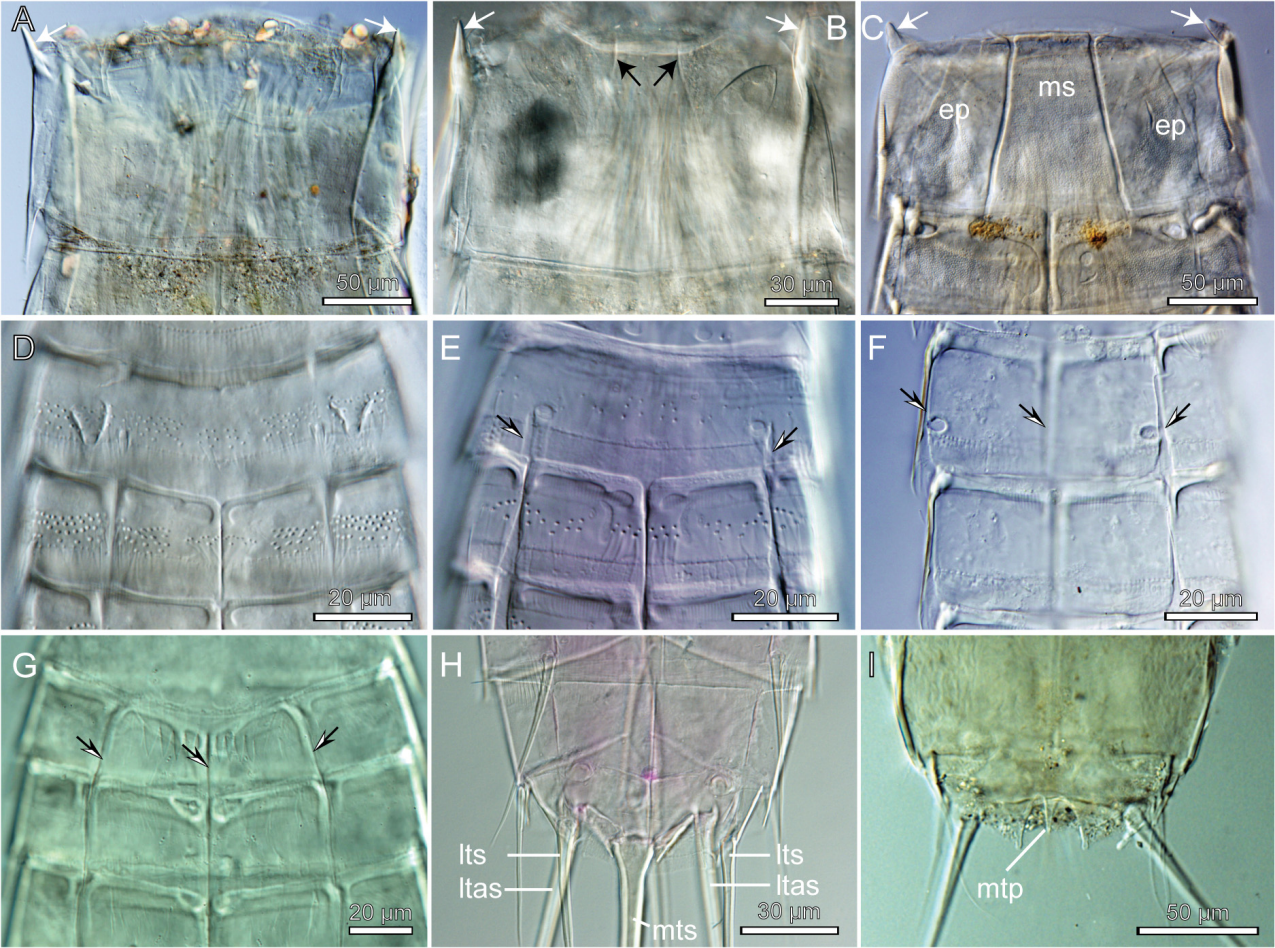
Light micrographs showing selected morphological character states for characters 14 to 42. A. Ventral view of segment 1 in *Mixtophyes abyssalis*, showing an undifferentiated sternal plate (character 16, state 0), and lateral projections at the anterior segment margin (white arrows) (character 14, state 1). B. Ventral view of segment 1 in *Paracentrophyes anurus*, showing a partly differentiated sternal plate (partial fissure marked with black arrows) (character 16, state 1), and lateral projections at the anterior segment margin (white arrows) (character 14, state 1). C. Ventral view of segment 1 in *Pycnophyes greenlandicus*, showing fully differentiated midsternal (ms) and episternal (ep) plates (character 16, state 2). Note also the lateral projections at the anterior segment margin (white arrows) (character 14, state 1). D. Ventral view of segments 2 and 3 in *Echinoderes astridae*, showing segment 2 forming a completely closed ring (character 19, state 0). E. Ventral view of segments 2 and 3 in *Meristoderes macracanthus*, showing partly differentiated lateral fissures (black/white arrows) on segment 2, but no midsternal fissure (character 19, state 1). F-G. Ventral view of segments 2 and 3 in *Fissuroderes sorenseni* (F) and *Polacanthoderes martinezi* (G), showing completely developed lateral and midsternal fissures (black/white arrows) on segment 2, and hence fully differentiated sternal plates (character 19, state 3). H. Ventral view of segments 10 and 11 in *Wollunquaderes majkenae*, showing midterminal (mts), lateral terminal (lts) and lateral terminal accessory spines (ltas) (character 38, state 1 and character 41, state 1). I. Ventral view of segments 10 and 11 in *Paracentrophyes quadridentatus*, showing its minute midterminal process (mtp) (character 41, state 0 and character 42, state 1).

The idea that echinoderid phylogeny can be understood through the differentiation of tegumental plates in segment 2 is in many ways logic, but we also see some problems, and things might not be as simple as they appear. For instance, segment 2 in species of *Meristoderes* (Fig 6E) is considered to represent a transitional stage between a condition with segment 2 being composed of one tergal and two sternal plates (Fig 6F and 6G), and segment 2 forming a closed ring (Fig 6D). This intermediate condition is perfectly expressed in the genus’ type species, *M*. *macracanthus*, that shows partly developed lateroventral fissures on the posterior half of the segment and a very weak indication of an intracuticular midventral line or fissure (Fig 6E). An almost identical condition is expressed in *M*. *galatheae* [35]. However, after these two species were described and assigned to the genus, additional *Meristoderes* species have been discovered and revealed that the development of segment 2 fissures, and hence the partial plate differentiations may differ greatly among the species. For instance, the partial lateroventral fissures in *M*. *herranzae* and *M*. *imugi* extent almost to the anterior margin of segment 2, opposed to the condition in *M*. *macracanthus* and *M*. *galatheae* where the fissures are restricted to the posterior part of the segment [36]. Furthermore, the partial fissures in *M*. *elleae* are reduced to extremely weak indications on the surface on the cuticle, whereas the fissures in *M*. *glaber* are so well-developed that they form actual tergosternal junctions [36]. This variation in composition of segment 2 could be systematically problematic because it might indicate a possible paraphyly of the genus. If we expect the composition of segment 2 in *Meristoderes* to represent a transitional stage towards either fully differentiated plates, or alternatively, towards a closed ring, some of the conditions we can observe in the different species of *Meristoderes*, would have to be more apomorphic than others. For instance, if the transition is going towards fully differentiated tegumental plates, the condition in *M*. *glaber* would be more apomorphic than in any other species of *Meristoderes*, and *M*. *glaber* would hence be closer to species of *Cephalorhyncha*, *Polacanthoderes* and *Fissuroderes*. Alternatively, if the transition is going towards a segment 2 forming a closed ring, as expressed in species of *Echinoderes*, a species like *M*. *elleae* with only extremely weak indications of plate differentiations would be closer to *Echinoderes* than to other species of *Meristoderes*. Under both scenarios, the monophyly of *Meristoderes* is left as questionable.

The differentiation of segment 2 also poses systematic challenges outside *Meristoderes*. According to the genus diagnosis (e.g. [3], [37]) the crown character for the diverse genus *Echinoderes* is segment 2 forming a closed ring (Fig 6D). However, some species of *Echinoderes* actually show indications of an intracuticular midventral fissure in segment 2. Such indications are present at least in *E*. *angustus*, *E*. *aquilonius*, and *E*. *eximus* (see [38]), in *E*. *intermedius* and *E*. *truncatus* (see [35]), and in *E*. *aureus* (see [39]). Furthermore, midventral intracuticular thickenings (apodemes) in segment 2 are reported from *E*. *tubilak* [38], and *E*. *setiger* [3]. Such intracuticular thickenings typically occur around plate articulations, hence the thickenings could indicate a beginning plate differentiation, or alternatively, be the last trace of two plates that have fused. Except for *E*. *setiger*, the first author has had the opportunity to examine type material for all these species, and it can be confirmed that these midventral structure appear consistently, and do not resemble artifacts. If species of *Echinoderes* show indications of partial plate differentiations in segment 2, this would question the most important character of the genus – namely segment 2 forming a complete, closed ring.

In general, the echinoderid species display a mosaic of characters, and it remains to be tested which characters provide phylogenetic significant information, and which are homoplastic. Especially characters that occur across the generic borders should be subject of special attention. These characters include for instance glandular cells with funnel-shaped subcuticular structures, that have been reported from species of *Fissuroderes* and *Echinoderes* [34], [40], glandular cell outlets type 2, that appear in many, but not all species of the genera *Fissuroderes*, *Meristoderes*, and *Echinoderes* (e.g., [34], [36], [41], [42], [43]), and the tergal plate of segment 11, that in some species of *Fissuroderes*, *Meristoderes*, *Cephalorhyncha*, and *Echinoderes* is subdivided by a middorsal fissure [34], [35], [41]. If some of these characters are homologous across the genera, this could also have a significant influence on our understanding of echinoderid phylogeny.

It is beyond the scope of this paper to solve echinoderid phylogeny, as this would require a much denser echinoderid taxon sampling than the one we can present here. However, we would like to highlight this challenge as one of the greatest future tasks in kinorhynch phylogeny. Even though the monophyly of *Echinoderes* has been questioned only rarely in any modern contribution, we would like to stress the need for proper phylogenetic analysis of this extremely specious genus, and its closest allies. Furthermore, we would also draw attention to the fact that monophyly basically can be questioned for every single echinoderid genus, which stresses the need for a revision and a detailed phylogenetic analysis even more.

#### The position of *Campyloderes*

In all analyses, morphological (Fig 1), molecular (Fig 2) as well as combined morphological and molecular (Fig 3), *Campyloderes* appears as an isolated lineage within Cyclorhagida. This special status of *Campyloderes* has already been demonstrated in previous analyses of kinorhynch phylogeny, based on analyses of molecular sequence data [19], [20], but for the first time, this is also confirmed by analysis of morphological data.

Traditionally, *Campyloderes* has been united with *Condyloderes* and *Centroderes*, in the family Centroderidae [3], [15], [17], [44]. Higgins [15] and Neuhaus [3] list a number of diagnostic characters for the family, but their value as synapomorphic characters for all three genera can all be questioned. The proposed diagnostic traits are listed below, followed by comments regarding their value as potential autapomorphies for Centroderidae:

1. Spindle- to cigar-shaped habitus – this habitus would apply to most cyclorhagid species, and cannot be considered autapomorphic for Centroderidae.
2. Presence of 14 trichoscalids – this is correct, but the occurrence of 14 trichoscalids appears to be common for most kinorhynchs, except *Dracoderes* and echinoderids, hence this trait cannot be autapomorphic for Centroderidae.
3. Outer oral styles fused laterally [with mouth cone] or with 1 or 2 elements – the character trait “outer oral styles fused laterally” refers to the absence of outer oral styles in *Campyloderes* (Fig 4C) (see also, e.g., [45], [46]). This trait is autapomorphic for the genus. Interestingly though, the outer oral styles in species of *Condyloderes* appear to be, if not lost or fused, then at least strongly reduced (Fig 4D) [47], but it is uncertain whether or not there is an evolutionary link between the lack of outer oral styles in *Campyloderes* and the reduction in *Condyloderes*. In the morphological analysis of the present study, these two conditions were coded as different stages in the same character, but the analysis did not unambiguously support the existence of a transformation series. The outer oral styles in species of *Centroderes* do not differ significantly from the styles in most other cyclorhagids (see e.g., [48], [49]).
4. Presence of 14 or 16 placids – 14 placids are present in species of *Campyloderes* only [45], [46], whereas species of *Centroderes* and *Condyloderes*, and apparently all other cyclorhagids have 16 placids. Hence, nothing in the number of placids supports a closer relationship between *Campyloderes* and the two other centroderid genera.
5. Placids with either (a) broad midventral placid + two narrow ventrolateral placids + other placids alternatingly narrow and broad, or (b) very broad midventral placid + other placids alternatingly narrow and broad – condition (a) applies to species of *Centroderes* and *Condyloderes* [47]–[49], whereas condition (b) applies to *Campyloderes* exclusively [45], [46]. Hence, this trait does not support *Campyloderes* as more closely related with neither *Centroderes* nor *Condyloderes*.
6. Segment 1 ring-like, segments 2 to 11 with one tergal and two sternal plates – this composition of trunk segments is very common and found within all major kinorhynch linages, hence it cannot be autapomorphic for Centroderidae.
7. Middorsal spines at least on segments 1–9; midterminal, lateral terminal and lateral terminal accessory spines present (occasionally also cuspidate spines) – these characters would also apply to several other cyclorhagid genera, inclusive *Antygomonas*, *Sphenoderes*, *Semnoderes*, *Wollunquaderes*, and *Tubulideres*, hence they cannot be autapomorphic for Centroderidae.
8. Midterminal spine either (a) much longer than lateral terminal spines, or (b) midterminal spine shorter than lateral terminal spines – condition (a) is present in species of *Centroderes* (as well as many other cyclorhagids), whereas condition (b) is present in species of *Condyloderes* and *Campyloderes*.

In summary, no clear autapomorphies appear to exist for Centroderidae, which, together with the results of the present, as well as previous [19], [20], analyses lead us to conclude that *Campyloderes* cannot be a part of Centroderidae. Instead, *Campyloderes* should be treated as a separate, basal cyclorhagid lineage, whereas *Centroderes* and *Condyloderes* appear to be sister groups (Fig 3). Hence, we would suggest that the family Centroderidae is maintained, but only with *Centroderes* and *Condyloderes* included.

As for *Campyloderes*, the analyses did not provide an unambiguous result regarding its exact position inside Cyclorhagida. The morphological analysis (Fig 1) placed *Campyloderes* in a trichotomy together with *Condyloderes* and a clade with all other cyclorhagids with midterminal spine, analysis of 18S rRNA (Fig 2C) and 18S rRNA + 28S rRNA of the complete taxon sampling (Fig 2B) placed *Campyloderes* as sister clade to Allomalorhagida, whereas analyses of 18S rRNA + 28S rRNA of the taxon sampling with some taxa omitted (Fig 2A) and of the combined morphological and molecular datasets (Fig 3) supported *Campyloderes* as the most basal cyclorhagid clade, and hence the sister taxon of Echinorhagata and a third clade with all remaining cyclorhagids. It would be impossible to draw any conclusion about the genus’ exact position from these incongruent results, but we find it obvious though that *Campyloderes* represents a separate, basal cyclorhagid lineage.

#### The third major cyclorhagid clade

Besides the two cyclorhagid lineages with *Campyloderes* and Echinoderidae, respectively, all remaining cyclorhagids are united in one large clade. This clade was supported in all analyses that include molecular sequence data (Figs 2 and 3), except those that generally failed to provide any resolution. An almost identical clade is recognized in the morphological analysis (Fig 1), however, with the difference that *Campyloderes* here is included in a basal trichotomy, and that *Franciscideres* and New Genus also appear within the clade.

Due to the congruent results from most analyses, inclusive BI of combined morphological and molecular data (Fig 3), we consider this clade as valid and well-supported, and would propose the name Kentrorhagata nom. nov. (from Greek *Kentron*, center, referring to the midterminal spine, + *rhagos*, slit opening, traditional suffix for kinorhynch orders) for the group. The strong support is reflected in all exclusively molecular trees, where the clade always appear with a bootstrap support value >95 and posterior probability of 1.00 (Fig 2). In the BI analysis of the combined morphological and molecular data (Fig 3) the posterior probability is slightly lower, pp = 0.86, which is due to homoplastic morphological characters.

Inside this clade, the results become a bit more obscure. Common for most analyses including molecular sequences is that *Centroderes* and *Condyloderes* branch off as sister taxa most basally within the clade (18S ML, 18S+28S BI, 18S+28S ML, morphology + molecular BI). The morphological analysis also supports a basal position of these two particular taxa, hence, we find it reasonable to assume that this result is reliable. Also *Triodontoderes anulap* and the four species of *Zelinkaderes* form a monophyletic group within Kentrorhagata, and hence support monophyly of Zelinkaderidae. The remaining taxa in the clade, represented by the genera *Antygomonas*, *Semnoderes*, *Sphenoderes*, *Wollunquaderes* and *Tubulideres* in the molecular analyses, and, in addition, *Cateria* in the combined morphological and molecular analysis, form various random topologies in the different trees, and not even generic monophyly is always recognized. We do not consider the relationships between these taxa as reliable results. Instead, they only reflect the need for a more diverse and comprehensive molecular sampling. Thus, the only conclusion that can be made is that we find support for monophyletic Zelinkaderidae and Centroderidae (with *Campyloderes* excluded from the latter though).

Among taxa within this clade, we will only address a single one, namely *Cateria*. Whereas most taxa are represented by molecular as well as morphological data, only morphological information was available for *Cateria*. This makes its position within this large cyclorhagid clade questionable, because its phylogenetic position may be difficult to establish with morphology only. *Cateria* has always, with its highly aberrant morphology, been considered one of the most peculiar kinorhynch genera. Gerlach [50] originally assigned Cateriidae to the Conchorhagata, indicating a putative relationship with *Semnoderes*. However, both Higgins [15] and Adrianov and Malakhov [17] preferred to assign *Cateria* to its own, monogeneric suborder, Cryptorhagae, within Cyclorhagida, indicating that it was difficult to point out any obvious close relatives. Quite recently the new genus and species *Franciscideres kalenesos* was described from Brazil [19], and for the first time, a species of another genus showed clear similarities with species of *Cateria*. Dal Zotto et al. [19] listed several potential synapomorphies for *Cateria* and *Franciscideres*, and suggested a close relationship (many of these traits are also present in the yet undescribed taxon “New Genus”). In the morphological analysis (Fig 1), *Cateria* and *Franciscideres* do not branch out together, but at least they both appear in the same, unresolved clade, together with species of *Zelinkaderes*, *Triodontoderes* and the new genus. This clade is nested inside a larger clade that unites all cyclorhagids with midterminal spine. However, a novel and interesting input from the molecular analyses – found in the present study, but also from previous [19], [20] – is that *Franciscideres* and New Genus actually are more closely related with the traditional homalorhagid taxa, and that they hence do not belong to this cyclorhagid clade. This leaves us somehow undecided regarding the position of *Cateria*. According to Dal Zotto et al. [19] *Franciscideres* and *Cateria* are closely related, but apparently the morphological evidence is not strong enough in itself to let *Cateria* follow *Franciscideres* when molecular sequence data places it inside Allomalorhagida. Hence, we can at present only speculate how molecular data from *Cateria* would affect the topology of the tree. However, we do not find it unlikely that it would also point *Cateria* in an allomalorhagid direction, and closer to *Franciscideres* and New Genus.

### A new kinorhynch classification

Even though the results of the present analyses are not conclusive at all levels, we have obtained so much novel information, and had so many results from previous analyses confirmed, that a new kinorhynch classification needs to be proposed. Our approach has been to maintain current names whenever possible. Hence, if only a single or very few taxa have been relocated, we prefer to maintain the current names, and simply just redefine the group. Also groupings that could not be confirmed in the present analysis, due to low resolution or low support measures will be maintained. Oppositely, obviously para- or polyphyletic groups will be rejected, and names for new clades will be proposed if their composition differs considerably from present days’ classification. The classification will be proposed with emphasis on the results of the BI analysis of the combined morphological and molecular datasets, but with preference for groups that appear congruently in results from other analyses as well. The new classification is proposed in Table 3.

**Table 3:** The new kinorhynch classification.

Class	Order	Family	Genus
Allomalorhagida	-	Dracoderidae Higgins & Shirayama, 1990	*Dracoderes* Higgins & Shirayama, 1990
nom. nov.	-	Franciscideridae Fam. nov.	*Franciscideres* Dal Zotto et al., 2013
			New Genus Yamasaki in prep.
	-	Pycnophyidae Zelinka, 1896	*Kinorhynchus* Sheremetevskij, 1974
			*Pycnophyes* Zelinka, 1907
	-	Neocentrophyidae Higgins, 1983	*Mixtophyes* Sánchez et al., 2014
			*Neocentrophyes* Higgins, 1969
			*Paracentrophyes* Higgins, 1983
Cyclorhagida	Echinorhagata nom. nov.	Echinoderidae Bütschli, 1876	*Cephalorhyncha* Adrianov, 1999
comb. nov.			*Echinoderes* Claparède, 1863
			*Fissuroderes* Neuhaus & Blasche, 2006
			*Meristoderes* Herranz et al., 2012
			*Polacanthoderes* Sørensen, 2008
	Kentrorhagata nom. nov.	Antygomonidae Adrianov & Malakhov, 1994	*Antygomonas* Nebelsick, 1990
		Cateriidae Gerlach, 1956	*Cateria* Gerlach, 1956
		Centroderidae Zelinka, 1896	*Centroderes* Zelinka, 1907
			*Condyloderes* Higgins, 1969
		Semnoderidae Remane, 1929	*Semnoderes* Zelinka, 1907
			*Sphenoderes* Higgins, 1969
		Zelinkaderidae Higgins, 1990	*Triodontoderes* Sørensen & Rho, 2009
			*Zelinkaderes* Higgins, 1990
		incertae sedis	*Tubulideres* Sørensen et al., 2007
		incertae sedis	*Wollunquaderes* Sørensen & Thormar, 2010
	Xenosomata Zelinka, 1907	Campyloderidae Remane, 1929	*Campyloderes* Zelinka, 1907

We will also use the results of the present study to propose a long needed correction of the taxonomic levels that are used in kinorhynch classification. Traditionally the two kinorhynch main clades Cyclorhagida and the now rejected Homalorhagida have been assigned order rank, which is a remain dating back to times where most minor invertebrate groups were lumped together in the obviously polyphyletic phylum ‘Aschelminthes’ (see e.g., [51]). However, as most general metazoan classifications today assign phylum rank to Kinorhyncha, it appears logical to assign class rank to Cyclorhagida and Allomalorhagida, and consequently assign order rank to the cyclorhagid groups Echinorhagata nom. nov., Kentrorhagata and Xenosomata (see Table 3). We would encourage other systematic and taxonomic researchers to follow this classification and these ranks in the future.

### Morphological character evolution within Kinorhyncha

Bayesian inference of the combined morphological and molecular datasets resulted in a tree (Fig 3) on which the morphological character evolution within Kinorhyncha can be explored. A summary with selected and significant autapomorphies for the various clades, as well as selected character transformations that appears equivocally, are listed in Table 4.

**Table 4:** Morphological character evolution in Kinorhyncha.

Clade	Character	Plesiomorphic condition	Apomorphic condition
Kinorhyncha	Size of outer oral styles	N/A	Outer oral styles of similar sizes
	Composition of outer oral styles	N/A	Rigid and articulated
	Separation of outer oral styles and mouth cone	N/A	Oral styles attach at proximal join only
	Number of trichoscalids	N/A	14 trichoscalids
	Attachment of trichoscalids	N/A	Trichoscalids attach directly on introvert
	Number of placids in neck	N/A	Equivocal: Four equally best states exist for this character: 6,7, 9 or 16 placids
	Attachment of placids	N/A	Placids attach through joint
	Composition of first segment	N/A	Complete ring
	Transition through formation of sternal plates in segment 2	N/A	Sternal plates fully differentiated
	Composition of third and fourth segment	N/A	One tergal and two sternal plates
	Composition of seventh to tenth segment	N/A	One tergal and two sternal plates
	Composition of terminal segment	N/A	One tergal and two sternal plates
	Overall distribution of middorsal spines	N/A	Spines can occur on any segment from segment 1 to 11
	Overall distribution of lateroventral spines	N/A	Spines can occur on any segment from segment 1 to 10
	Males with penile spines	N/A	Equivocal: Either autapomorphic for clade, with secondary loss in Kentrorhagata and Campyloderidae, or convergently evolving within clade, at Allomalorhagida and Echinoderidae
	Lateral terminal accessory spines in one or both sexes	N/A	Equivocal: Two equally best optimizations exist for this character
	Lateral terminal spines	N/A	Present
	Midterminal spine	N/A	Equivocal: See under Cyclorhagida
Allomalorhagida	Symmetry of closing apparatus	Radial symmetrical	Bilateral symmetrical
*Dracoderes*	Size of outer oral styles	Outer oral styles of similar sizes	Size of outer oral styles alternate between larger and smaller ones
	Number of trichoscalids	14 trichoscalids	9 trichoscalids
	Arrangement of dorsal spines	Aligned middorsally	Alternatingly laterally displaced
	Overall distribution of lateroventral spines	Spines can occur on any segment from segment 1 to 10	Spines restricted to segments between segment 6 and 9
Clade: Franciscideridae + Neocentrophyidae + Pycnophyidae	Anterior margin of first trunk segment with lateral projections	Absent	Equivocal: Either autapomorphic for clade, with secondary loss in Franciscideridae, or convergently evolving within clade, at Neocentrophyidae and Pycnophyidae
	Composition of first segment	Complete ring	Equivocal: Either plesiomorphic condition is retained, or one tergal and one broad sternal plate, eventually partially or fully differentiated into mid- and episternal plates develop
	Differentiation of sternal plate in taxa with one tergal and broad sternal plate	N/A	Equivocal: Either sternal plate partially subdivided or sternal plate differentiated into episternal and midsternal plates
	Trunk with non-articulated, middorsal structures	Absent	Equivocal: Middorsal non-articulated structures either autapomorphic for clade, with secondary loss in Franciscideridae, or convergently evolving within clade, at Neocentrophyidae and Pycnophyidae
	Trunk with setae	Absent	Equivocal: Setae either autapomorphic for clade, with secondary loss in Franciscideridae, or convergently evolving within clade, at Neocentrophyidae and Pycnophyidae
	Spines restricted to segments 10 and 11	Absent	Equivocal: Restriction of spines to segments 10 and 11 is either autapomorphic for clade, with secondary loss in Franciscideridae, or convergently evolving within clade, at Neocentrophyidae and Pycnophyidae
Franciscideridae	Neck region forms segment-like ring	Absent	Present
	Placids in neck	Present	Absent
Neocentrophyidae	Size of outer oral styles	Outer oral styles of similar sizes	Size of outer oral styles alternate between larger and smaller ones
	Midterminal process	Absent	Present
Pycnophyidae	Composition of outer oral styles	Rigid, articulated	Soft, non-articulated
	Sexual dimorphism expressed as presence of ventromedial tubes on segment 2 in males	Absent	Present
Cyclorhagida	Midterminal spine	Absent	Equivocal: Midterminal spine either autapomorphic for clade, with secondary loss in Echinorhagata, or convergently evolving within clade, at Kentrorhagata and Xenosomata
Xenosomata/Campyloderidae	Number of placids in neck	16 placids	14 placids
	Separation of outer oral styles and mouth cone	Oral styles attach at proximal join only	Oral styles completely fused with mouth cone
	Segment 1 with pair of extraordinary long lateroventral spines situated next to pair of very short ventrolateral spines	Absent	Present
Clade: Kentrorhagata + Echinoderidae	No apparent synapomorphies		
Echinorhagata/Echinoderidae	Number of trichoscalids	14 trichoscalids	6 trichoscalids
	Overall distribution of middorsal spines	Spines can occur on any segment from segment 1 to 11	Spines restricted to segments between segment 4 and 8
	Gender determined presence of lateral terminal accessory spines	Present in both sexes	Present in females only
Kentrorhagata	Cuspidate spines	Absent	Present

The table shows selected character transformations for different nodes and clades.

#### The kinorhynch ground pattern

In general, a long list of character transformations appears at the root of Kinorhyncha. Most of these characters would be inapplicable for the outgroup taxa, but still it makes sense to consider most of these characters as autapomorphic for Kinorhyncha and part of the kinorhynch ground pattern. These characters include the presence of rigid and articulating outer oral styles of similar lengths, attaching proximally to the mouth cone; introvert with 14 trichoscalids that attach directly on the introvert; neck composed of placids, articulating with the anterior margin of segment 1 along a distinct joint; a segmented trunk, with the first segment consisting of a complete ring, and the following ten segments consisting of one tergal and two sternal plates; dorsal and lateroventral spines present, and able to occur on all trunk segments (i.e. the occurrence of spines is not being restricted to certain segments); and lateral terminal spines present. Four important characters could unfortunately not be fully optimized, because several, equally parsimonious options were available. One character regards the original number of placids in the neck. No less than four, equally parsimonious solutions exist, namely a ground pattern with 6, 7, 9 or 16 placids. The difficulties with the optimization occur because even though the number of placids in cyclorhagids always appears to be fixed at 16, the number differs among species of Allomalorhagida. If we expect that kinorhynchs evolved from a priapulid-like ancestor and perhaps a fossil scalidophoran *Markuelia*-like ancestor, we would expect the head and neck region to show a radial symmetrical pattern. Based on this assumption, we find it most likely that the cyclorhagid number, i.e. 16 placids, was part of the kinorhynch ground pattern, and that the number of placids among species of Allomalorhagida got reduced when the symmetry patterns of the neck and the closing apparatus for head got modified from radial symmetrical to bilateral symmetrical. This is also supported by developmental observations in the homalorhagid *Paracentrophyes* [52], showing that the number of placids is reduced during juvenile development. Hence, we would tend to support that the kinorhynch ground pattern show 16 placids, even though it should be stressed that this is not unambiguously supported by our data. Another equivocal optimization regards the presence of penile spines that either evolved convergently at the bases of Allomalorhagida and Echinoderidae, or alternatively be a part of the kinorhynch ground pattern, but subsequently got lost in Kentrorhagata and Campyloderidae. We find it difficult to favor any of the two options. Also the evolution of lateral terminal accessory spines and midterminal spines is difficult to optimize unambiguously. According to the analysis, evolution of lateral terminal accessory spines is equivocal because it could either be a kinorhynch ground pattern character that subsequently was lost at the base of Allomalorhagida and *Condyloderes*, or alternatively evolved at the base of Cyclorhagida, and subsequently were lost at the base of *Condyloderes*. Again, it is impossible to point out one option with certainty, and in this particular case we find that both parsimonious options are equally likely. Almost the same options exist for the midterminal spine. It can either be a kinorhynch ground pattern character that subsequently was lost at the branches leading to Allomalorhagida and Echinoderidae, it could have convergently evolved in Kentrorhagata and Campyloderidae, or it could be a cyclorhagid autapomorphy that subsequently was lost at the base of Echinoderidae. We find it likely that either the first or the third option is the correct one. Convergent evolution of a midterminal spine is unlikely, and even though it is missing in adult specimens of Echinoderidae, we actually find midterminal spines in juveniles [53], [54], i.e., the genetic capability of forming a midterminal spine must be present in species of Echinoderidae also. Midterminal outgrowths also exist in juveniles of, e.g., *Pycnophyes* and *Paracentrophyes*. Here they appear as processes (i.e., direct cuticular extensions from the surface cuticle) rather than spines [52], [55], but based on ontological observations Neuhaus [3], [55] has suggested that midterminal processes in juvenile homalorhagids may represent structures that are homologous with midterminal spines. This would suggest that the genetic capability of forming a midterminal spine existed at the base of all kinorhynchs, whereas the presence of an articulating midterminal spine in adults would be a plesiomorphy for Cyclorhagida or an autapomorphy for Kentrorhagata.

#### Character transformations and synapomorphies for Allomalorhagida

**Fig 7:**
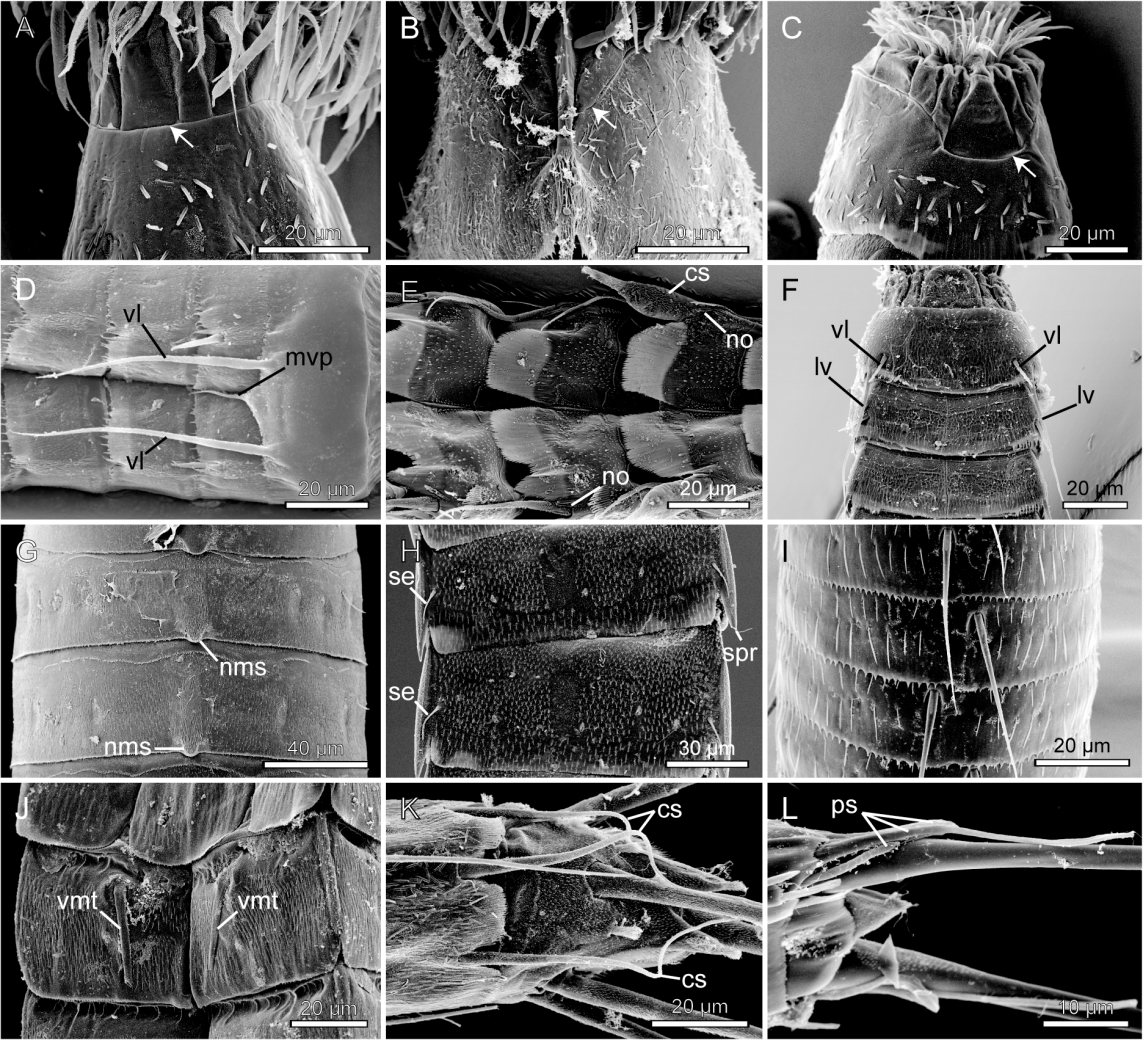
Scanning electron micrographs showing selected morphological character states for characters 17 to 37. A. Neck and segment 1 of *Antygomonas paulae* in ventrolateral view, showing the slightly sinuate anterior segment margin (arrow) (character 17, state 1). B. Neck and segment 1 of *Semnoderes armiger* in dorsal view, showing the deep middorsal incision in the anterior segment margin (arrow) (character 17, state 2). C. Neck and segment 1 of *Sphenoderes poseidon* in ventral view, showing the deep midventral incision in the anterior segment margin (arrow) (character 17, state 2). D. Segments 1 to 4 of *Centroderes spinosus* in ventral view, showing a straight anterior margin of the first segment (character 17, state 0), spinose midventral process (mvp) (character 23, state 1) and extraordinary long ventrolateral spines (vl) (character 33, state 1) present in the posterior margin. E. Segments 5 to 7 of *A*. *paulae* in ventral view, showing deep lateroventral notches (no) in the posterior segment margins (character 24, state 1). Note also the cuspidate spine (cu) (character 34, state 1). F. Segments 1 to 3 of *Campyloderes* cf. *vanhöffeni* in ventral view, showing extraordinary long lateroventral spines (lv) close to much shorter ventrolateral spines (vl) (character 32, state 1). G. Mid- and subdorsal parts of segments 3 to 5 in *Pycnophyes* sp., showing small non-articulated middorsal structures (nms) hardly projecting beyond the posterior segments margins (character 26, state 1). H. Segments 7 and 8 of *Paracentrophyes anurus* in lateral view, showing large non-articulated middorsal structures extended into spinose processes (spr) projecting beyond the posterior segment margins (character 26, state 0). Note also the small lateroventral setae (se) (character 27, state 1). I. Segments 3 to 5 of *Dracoderes abei* in dorsal view, showing alternatingly laterally displaced dorsal spines (character 30, state 1). J. Segment 2 of *Pycnophyes kielensis* in ventral view, showing male specific ventromedial tubes (vmt) (character 35, state 1). K. Segment 11 of *Triodontoderes anulap* in dorsal view, showing male specific crenulated spines (cs) in laterodorsal and middorsal positions (character 36, state 1). L. Segment 11 of *D*. *abei* in ventrolateral view, showing male specific penile spines (ps) (character 37, state 1).

The newly erected class Allomalorhagida is basically only supported morphologically by a single autapomorphy, namely the modification of the neck and closing apparatus from being radial symmetrical to bilateral symmetrical. A radial symmetrical neck region exists among most cyclorhagids, except for species of *Semnoderes*, *Sphenoderes* and *Antygomonas*. However, the bilaterally contracting clamshell-like closing apparatus (Fig 7A, 7B and 7C) in these exceptional species differs considerably from the closing apparatus in species of Allomalorhagida that contracts dorsoventrally. In species of Pycnophyidae and Neocentrophyidae, the bilateral symmetry is very conspicuous, and the placids are arranged as a dorsal and a ventral set. This arrangement is less conspicuous in species of *Dracoderes* (Fig 5C), although the short but wide placids, with cuticular foldings in between are still arranged in a pattern that is much closer to the arrangement in the pycnophyid and neocentrophyid species [32], [33]. The neck regions in *Franciscideres* and New Genus are radial symmetrical, but the neck regions in these species have generally been going through significant modifications, so that they appear as segment-like rings, basically without any placids at all, which is a unique condition among Kinorhyncha [19].

Autapomorphies for Dracoderidae are obviously the reduced number of trichoscalids (from 14 to 9) and the alternatingly laterally displaced middorsal spines (Fig 7I). It has been suggested that the alternating lengths of the outer oral styles, present in Dracoderidae (Fig 4B) and Neocentrophyidae, could be synapomorphic for the two groups as well [32], [33], but this is not supported by the present analysis. Instead, the analysis suggests that this alternation in lengths evolved independently within the two clades.

The clade consisting of Franciscideridae, Neocentrophyidae, and Pycnophyidae is supported by several potential synapomorphies, but the character optimization is ambiguous for all of them, hence they are somehow questionable. The characters include: a) presence of lateral projections at anterior margin of first trunk segment (Fig 6A, 6B and 6C); b) first trunk segment composed of a tergal plate and a sternal plate that eventually is partially (Fig 6B) of fully (Fig 6C) differentiated into epi- and midsternal plates; c) presence of middorsal processes (Fig 7G and 7H) and lateroventral setae (Fig 7H); and d) restriction of spines to the very posterior segments. All of these characters represent what we would think of as typical “homalorhagid” traits, and hence their optimization depends on if we consider species of Franciscideridae as highly modified, or whether *Franciscideres* represents a more basal, conserved morphology, which would affect that many of these character traits would have to be convergently evolved within Neocentrophyidae and Pycnophyidae. A third option is of course that *Franciscideres* has been misplaced and should appear as sister taxon to Neocentrophyidae and Pycnophyidae (= Homalorhagida), but since this option has not been supported in a single analysis, we cannot really consider it, and at the same time stay true with our results.

The three remaining Allomalorhagida families are supported by rather uncontroversial characters. Franciscideridae is supported by its special neck region, forming a segment-like ring (Fig 5A), and then the reduction of placids. Pycnophyidae is supported by the modification of their outer oral styles that change from articulated and rigid to unarticulated and soft (Fig 4E and 4F), and by the presence of sexually dimorphic ventromedial tubes on segment 2 in males (Fig 7J) (not present in all species of *Pycnophyes* though). Both are typical and relatively unproblematic autapomorphies for Pycnophyidae. Inside Pycnophyidae, no analyses are able to support monophyletic *Pycnophyes* and *Kinorhynchus*. If we look at the character transformations through the tree, the presence of lateral terminal spines would actually be plesiomorphic for Pycnophyidae, meaning that the lack of such spines in *Kinorhynchus*, which after all is the most important diagnostic character for the genus, actually could be considered autapomorphic. However, since no other characters support the distinction between the two genera, it must be up to future studies to test their eventual polyphyly or paraphyly.

#### Character transformations and synapomorphies for Cyclorhagida

The major clade Cyclorhagida has existed in kinorhynch literature for more than a century [56]. The clade is also recovered in most analysis, but interestingly, it is only supported by a single potential morphological character, namely the presence of midterminal spine, and as discussed above, this character cannot even be optimized unambiguously. However, despite the low morphological support, we believe that the clade is monophyletic, which is also supported congruently by most analyses.

Also the large clade Kentrorhagata is characterized by low morphological support. Again, only a single potential autapomorphy appears, namely the development of cuspidate spines (Fig 7E).

In the BI tree based on combined morphological and molecular data (Fig 3), Kentrorhagata and Echinorhagata appear as sister clades. However, we do not find any morphological support for this clade. We will not reject the clade as a possibility, but the lacking support stresses that the interrelationships between the three cyclorhagid main lineages remain unresolved. Even though we find monophyletic Cyclorhagida, accommodating monophyletic Kentrorhagata, Xenosomata, and Echinorhagata in most analyses, the relationships between the three clades differ between the different analyses. This indicates that we are close to the limit of what our data can resolve, and therefore, we hesitate to make any final conclusion about the relationships between these three major cyclorhagid clades. Hence, for now, we will just conclude that our most data-loaded analysis supports a sister-group relationship between Kentrorhagata and Echinoderidae, and then let more comprehensive future studies test whether this result is correct.

The two remaining major clades, Xenosomata and Echinorhagata, are rather unproblematic. Xenosomata is supported by typical diagnostic *Campyloderes* characters (see also [45], [46]) such as reduced placid number, from 16 to 14; outer oral styles being lost or fused with mouth cone; and the presence of extraordinary long lateroventral spines on segment 1. Echinorhagata is likewise supported by typical echinoderid autapomorphies, inclusive the number of trichoscalids that is reduced from 14 to 6; restriction of lateral accessory spines to females only; and an apparent restriction of the middorsal spines to appear only on segments 4 to 8.

## Conclusions

The phylogeny of Kinorhyncha was analyzed using molecular and morphological data, separately and in combination. The analyses showed that our data provided some clear signal, but also had its limitations. One limitation for the morphological data and analyses was the problems related to the outgroup comparison. Even though priapulids appear as one of the most likely candidates for a kinorhynch sister clade, the two animal groups are still so fundamentally different, that unambiguous character polarization hardly can be obtained for any character. Hence, in studies approaching general kinorhynch phylogeny, inclusive phylogeny near the kinorhynch root, morphological characters would probably always have to be accompanied by molecular data, in order to set the root. Limitations of the two molecular loci, 18S rRNA and 28S rRNA, regarded their ability to resolve relationships between more closely related taxa. Even though we are aware that taxonomic levels are completely arbitrary, there seemed to be a threshold around the family level, and that relationships above this level could only be poorly resolved with the chosen loci. Hence, our recommendation for future studies would be to use a more diverse selection of molecular markers, or alternatively a transcriptomic approach, to resolve the relationships within the major clades that were recognized in the present study.

The most significant and congruent results were obviously obtained in the more basal parts of the tree. Based on our analyses, we can conclude that Kinorhyncha can be subdivided into two major clades: Cyclorhagida and Allomalorhagida. The latter is a new assemblage of taxa that includes Dracoderidae, Franciscideridae, and the traditional homalorhagid taxa. We do not find support for monophyletic homalorhagids though, hence for now, we will have to reject this taxon as a taxonomic and phylogenetic unit. Likewise, we would like to draw attention to the apparent paraphyly to the species-rich, formerly homalorhagid genera *Pycnophyes* and *Kinorhynchus*. Even though our data does not allow us to draw any conclusions at subgeneric levels, we find it noteworthy that the present, as well as all previous molecular analyses [19], [20], have failed to support monophyly for these two particular genera, and we would encourage future studies to focus on eventual revision of the Pycnophyidae.

Cyclorhagida accommodates three major lineages: Echinoderidae, Campyloderidae (with species of *Campyloderes* only), and the large clade Kentrorhagata, that – except from species of *Campyloderes* – includes all species with a midterminal spine. The exact relationship between these three clades should be explored further in future studies. Otherwise, we would recommend that future cyclorhagid studies focus on the internal relationships within the two large clades Echinoderidae and Kentrorhagata. After several attempts, in this as well as previous studies, we still have very little information about the phylogenetic pathways within Echinoderidae. These should be explored further, especially because we identify some signs indicating that the large genus *Echinoderes* could be paraphyletic. Hence, a revision of *Echinoderes* and a general study of the phylogenetic pathways within Echinoderidae are highly needed. Kentrorhagata also attracts special attention. Even though some of the taxa forming this clade have been grouped together previously in different phenetic classifications, this is the first time where these taxa are united in a monophyletic group. The monophyly of the group seems to be well-supported by most data, but the internal relationships are still unresolved, and should be addressed in studies with improved molecular and taxonomic sampling.

A final question to address in future studies is the position of *Cateria*. The results of the present analyses suggest that the genus should be nested inside Kentrorhagata, whereas previous morphological studies strongly suggest a closer relationship with Franciscideridae. In the present analyses *Cateria* has only been represented by morphological data, and it would be extremely interesting to test if its position inside Kentrorhagata is supported by molecular data also, or whether such data would bring *Cateria* closer to Franciscideridae. Hence, acquisition of molecular sequence data from representatives of *Cateria* should be given high priority.

Conclusively, the present study has brought us a big step closer to an understanding of the basal phylogenetic pathways within Kinorhyncha. From the present study we are able to predict a broad range of kinorhynch ground pattern characters, and explain the early evolution within the group. Yet, many questions are left unanswered when we move upwards in the kinorhynch tree. Future studies will hopefully provide us with more detailed information about the relationships within the kinorhynch main clades.

## Supporting Information

S1 File Appendix A: The morphological characters. (DOCX)

S2 File Appendix B: The morphological matrix. (DOCX)

## References

[1] Dujardin F. Sur un petit animal marin, l’Echinodère, formant un type intermédiaire entre les Crustacés et les Vers. Ann Sci naturelles (3) Zoologie 1851;15: 158–173.

[2] Claparède ARE. Zur Kenntnis der Gattung *Echinoderes* Duj Beobachtungen über Anatomie und Entwicklungsgeschichte wirbelloser Thiere an der Küste von Normandie angestellt. Leipzig: Verlag von Wilhelm Engelmann; 1863.

[3] Neuhaus B. 5. Kinorhyncha (= Echinodera) In: Schmidt-Rhaesa A, editor. Handbook of Zoology. Gastrotricha, Cycloneuralia and Gnathifera. Nematomorpha, Priapulida, Kinorhyncha, Loricifera. Berlin/Boston: De Gruyter; 2013 pp. 181–348.

[4] Sørensen MV. Phylum Kinorhyncha. Zootaxa 2013;3703: 63–66.

[5] Sánchez N, Pardos F, Sørensen MV. A new kinorhynch genus, *Mixtophyes* (Kinorhyncha: Homalorhagida), from the Guinea Basin deep-sea, with new data on the family Neocentrophyidae. Helgoland Mar Res. 2014;68: 221–239.

[6] Nielsen C. Animal Evolution. Interrelationships of the living phyla. 3rd ed. Oxford: Oxford University Press; 2012.

[7] Hejnol A, Obst M, Stamatakis A, Ott M, Rouse GW, Edgecombe GD, et al Assessing the root of bilaterian animals with scalable phylogenomic methods. Proc R Soc, Ser B. 2009;276: 4261–4270. 10.1098/rspb.2009.0896 PMC2817096 19759036

[8] Paps J, Baguñà J, Riutort M. Bilaterian phylogeny: A broad sampling of 13 nuclear genes provides a new Lophotrochozoa phylogeny and supports a paraphyletic basal Acoelomorpha. Mol Biol Evolut. 2009;26: 2397–2406. 10.1093/molbev/msp150 19602542

[9] Borner J, Rehm P, Schill RO, Ebersberger I, Burmester T. A transcriptome approach to ecdysozoan phylogeny. Mol Phylogenet Evolut. 2014;80: 79–87. 10.1016/j.ympev.2014.08.001 25124096

[10] Neuhaus B, Higgins RP. Ultrastructure, biology and phylogenetic relationships of Kinorhyncha. Integ Comp Biol. 2002;42: 619–632. 10.1093/icb/42.3.619 21708758

[11] Park J-K, Rho HS, Kristensen RM, Kim W, Giribet G. First molecular data on the phylum Loricifera—an investigation into the phylogeny of Ecdysozoa with emphasis on the positions of Loricifera and Priapulida. Zool Sci. 2006;23: 943–954. 17189906 10.2108/zsj.23.943

[12] Sørensen MV, Hebsgaard MB, Heiner I, Glenner H, Willerslev E, Kristensen RM. New Data from an Enigmatic Phylum: Evidence from molecular sequence data supports a sister group relationship between Loricifera and Nematomorpha. J Zool Syst Evolut Res. 2008;46: 231–239.

[13] Zelinka C. Zur Kenntnis der Echinoderen. Zool Anz. 1907;32: 130–136.

[14] Higgins RP. Three new Kinorhynchs from the North Carolina coast. Bull. mar. Sci. Gulf Carribean 1964;14: 479–493.

[15] Higgins RP. Zelinkaderidae, a new family of cyclorhagid Kinorhyncha. Smithson Contrib Zool. 1990;500: 1–26.

[16] Adrianov AV, Malakhov VV. The phylogeny and classification of the class Kinorhyncha. Zoosyst Ross. 1996;4: 23–44.

[17] Adrianov AV, Malakhov VV. Cephalorhyncha of the World Ocean. Moscow: KMK Scientific Press; 1999.

[18] Sørensen MV. Phylogenetic analysis of the Echinoderidae (Kinorhyncha: Cyclorhagida). Org Div Evol. 2008;8: 233–246.

[19] Dal Zotto M, Di Domenico M, Garraffoni A, Sørensen MV. *Franciscideres* gen. nov.—a new, highly aberrant kinorhynch genus from Brazil, with an analysis of its phylogenetic position. Syst Biodiv. 2013;11: 303–321.

[20] Yamasaki H, Hiruta SF, Kajihara H. Molecular phylogeny of kinorhynchs. Mol Phylogenet Evolut. 2013;67: 303–310. 10.1016/j.ympev.2013.02.016 23454469

[21] Page RDM; 2001. NEXUS Data Editor for Windows. Software and documentation available at: http://taxonomy.zoology.gla.ac.uk/rod/NDE/nde.html. Accessed 13 October 2014.

[22] Goloboff P, Farris JS, Nixon KC; 2003. TNT: Tree Analysis Using New Technology. Software and documentation available at: http://www.cladistics.com/aboutTNT.html. Accessed 13 October 2014.

[23] Katoh K, Toh H. Recent developments in the MAFFT multiple sequence alignment program. Brief. Bioinform. 2008;9: 286–298. 10.1093/bib/bbn013 18372315

[24] Katoh K, Toh H. Parallelization of the MAFFT multiple sequence alignment program. Bioinformatics 2010;26: 1899–1900. 10.1093/bioinformatics/btq224 20427515 PMC2905546

[25] Capella-Gutierrez S, Silla-Martinez JM, Gabaldon T. TrimAl: a tool for automated alignment trimming in large-scale phylogenetic analyses. Bioinformatics 2009;25: 1972–1973. 10.1093/bioinformatics/btp348 19505945 PMC2712344

[26] Tanabe AS. KAKUSAN: a computer program to automate the selection of a nucleotide substitution model and the configuration of a mixed model on multilocus data. Mol Ecol Notes 2007;7: 962–964.

[27] Stamatakis A. RAxML-VI-HPC: maximum likelihood-based phylogenetic analyses with thousands of taxa and mixed models. Bioinformatics 2006;22: 2688–2690. 16928733 10.1093/bioinformatics/btl446

[28] Silvestro D, Michalak I. raxmlGUI: a graphical front-end for RAxML. Org Div Evolut. 2012; 12: 335–337.

[29] Ronquist F, Huelsenbeck JP. MrBayes 3: Bayesian phylogenetic inference under mixed models. Bioinformatics 2003;19: 1572–1574. 12912839 10.1093/bioinformatics/btg180

[30] Maddison WP, Maddison DR; 2014. Mesquite: a modular system for evolutionary analysis. Version 3.01 http://mesquiteproject.org. Accessed 13 November 2014.

[31] Higgins RP, Shirayama Y. Dracoderidae, a new family of the cyclorhagid Kinorhyncha from the Inland Sea of Japan. Zool Sci. 1990;7: 939–946.

[32] Sørensen MV, Herranz M, Rho HS, Min W, Yamasaki H, Sánchez N, et al On the genus *Dracoderes* Higgins & Shirayama, 1990 (Kinorhyncha: Cyclorhagida) with a redescription of its type species, *D*. *abei*, and a description of a new species from Spain. Mar Biol Res. 2012;8: 210–232.

[33] Thomsen VG, Rho HS, Kim D, Sørensen MV. A new species of *Dracoderes* (Kinorhyncha: Dracoderidae) from Korea provides further support for a dracoderid-homalorhagid relationship. Zootaxa 2013;3682: 133–142. 25243279 10.11646/zootaxa.3682.1.6

[34] Neuhaus B, Blasche T. *Fissuroderes*, a new genus of Kinorhyncha (Cyclorhagida) from the deep sea and continental shelf of New Zealand and from the continental shelf of Costa Rica. Zool Anz. 2006;245: 19–52.

[35] Herranz M, Thormar J, Benito J, Sánchez N, Pardos F. *Meristoderes* gen. nov., a new kinorhynch genus, with the description of two new species and their implications for echinoderid phylogeny (Kinorhyncha: Cyclorhagida, Echinoderidae). Zool Anz. 2012;251: 161–179

[36] Sørensen MV, Rho HS, Min W, Kim D, Chang CY. Occurrence of the newly described kinorhynch genus *Meristoderes* (Cyclorhagida: Echinoderidae) in Korea, with the description of four new species. Helgoland Mar Res. 2013;67: 219–319.

[37] Higgins RP. The Atlantic barrier reef ecosystem at Carrie Bow Cay, Belize, II: Kinorhyncha. Smithson Contrib Mar Sci. 1983;18: 1–131.

[38] Higgins RP, Kristensen RM. Kinorhyncha from Disko Island, West Greenland. Smithson Contrib Zool. 1988;458: 1–56.

[39] Adrianov AV, Murakami, Shirayama Y. *Echinoderes aureus* n. sp. (Kinorhyncha: Cyclorhagida) from Tanabe Bay (Honshu) – first representative of the genus in the Pacific Ocean. Proc Biol Soc Wash. 2002;115: 205–216.

[40] Thormar J, Sørensen MV. Two new species of *Echinoderes* (Kinorhyncha: Cyclorhagida) from the Solomon Islands. Meiofauna Mar. 2010;18: 67–96.

[41] Sørensen MV, Rho HS, Min W, Kim D, Chang CY. An exploration of *Echinoderes* (Kinorhyncha: Cyclorhagida) in Korean and neighboring waters, with the description of four new species and a redescription of *E*. *tchefouensis* Lou, 1934. Zootaxa 2012;3368: 161–196.

[42] Herranz M, Pardos F. *Fissuroderes sorenseni* sp. nov. and *Meristoderes boylei* sp. nov.: First Atlantic recording of two rare kinorhynch genera, with new identification keys. Zool Anz. 2013;253: 91–111.

[43] Sørensen MV. First account of echinoderid kinorhynchs from Brazil, with the description of three new species. Mar Biodiv 2014;44: 251–274.

[44] Higgins RP. Indian Ocean Kinorhyncha: 1, *Condyloderes* and *Sphenoderes*, new cyclorhagid genera. Smithson Contr Zool. 1969;14: 1–13.

[45] Neuhaus B. Description of *Campyloderes* cf. *vanhoeffeni* (Kinorhyncha, Cyclorhagida) from the Central American East Pacific deep sea with a review of the genus. Meiofauna Mar. 2004;13: 3–20.

[46] Neuhaus B, Sørensen MV. Populations of *Campyloderes* sp. (Kinorhyncha, Cyclorhagida): One global species with significant morphological variation? Zool Anz. 2013;252: 48–75.

[47] Sørensen MV, Rho HS, Kim D. A new species of *Condyloderes* (Cyclorhagida, Kinorhyncha) from Korea. Zool Sci. 2010;27: 234–242. 10.2108/zsj.27.234 20192691

[48] Neuhaus B, Pardos F, Sørensen MV, Higgins RP. Redescription, morphology, and biogeography of *Centroderes spinosus* (Reinhard, 1881) (Kinorhyncha, Cyclorhagida) from Europe. Cah Biol Mar. 2013;54: 109–131.

[49] Neuhaus B, Pardos F, Sørensen MV, Higgins RP. New species of *Centroderes* (Kinorhyncha: Cyclorhagida) from the Northwest Atlantic Ocean, life cycle, and ground pattern of the genus. Zootaxa. 2014;3901: 1–69. 10.11646/zootaxa.3901.1.1 25543839

[50] Gerlach SA. Über einen aberranten Vertreter der Kinorhynchen aus dem Küstengrundwasser. Kieler Meeresforsch. 1956;12: 120–124.

[51] Hyman LH. The Invertebrates. Acanthocephala, Aschelminthes and Entoprocta The Pseudocoelomate Bilateria. Vol. 3 New York: McGraw-Hill; 1951.

[52] Neuhaus B. Postembryonic development of *Paracentrophyes praedictus* (Homalorhagida): neoteny questionable among the Kinorhyncha. Zool Scr. 1995;24: 179–192.

[53] Higgins RP. Two new species of *Echinoderes* (Kinorhyncha) from South Carolina. Trans Am Microsc Soc. 1977;96: 340–354.

[54] Sørensen MV, Jørgensen A, Boesgaard TM. A new *Echinoderes* (Kinorhyncha: Cyclorhagida) from a submarine cave in New South Wales, Australia. Cah Biol Mar. 2000;41: 167–179.

[55] Neuhaus B. Postembryonic development of *Pycnophyes kielensis* and *P* . *dentatus* (Kinorhyncha) from the North Sea. Microfauna Mar. 1993;8: 163–193.

[56] Zelinka K. Demonstration von Tafeln der *Echinoderes*-Monographie. Verh Dtsch Zool Gesell. 1896;6: 197–199.

[57] Dal Zotto M. *Antygomonas caeciliae*, a new kinorhynch from the Mediterranean Sea, with report of mitochondrial genetic data for the phylum. Mar Biol Res. 2015;in press. 10.1080/17451000.2015.1007872

[58] Sørensen MV. A new species of *Antygomonas* (Kinorhyncha: Cyclorhagida) from the Atlantic coast of Florida, USA. Cah Biol Mar. 2007;48: 155–168.

[59] Higgins RP. Taxonomy and postembryonic development of the Cryptorhagae, a new suborder for the mesopsammic kinorhynch genus *Cateria* . Trans Amer Microsc Soc. 1968;87: 21–39.

[60] Nebelsick M. Sensory spots of *Echinoderes capitatus* (Zelinka, 1928) (Kinorhyncha, Cyclorhagida). Acta Zool. 1992;73: 185–195.

[61] Nebelsick M. Introvert, mouth cone, and nervous system of *Echinoderes capitatus* (Kinorhyncha, Cyclorhagida) and implications for the phylogenetic relationships of Kinorhyncha. Zoomorphology 1993;113: 211–232.

[62] Higgins RP. Redescription of *Echinoderes dujardinii* (Kinorhyncha) with descriptions of closely related species. Smithson Contrib Zool. 1977;248: 1–26.

[63] Higgins RP. *Echinoderes gerardi* n. sp. and *E*. *riedli* (Kinorhyncha) from the Gulf of Tunis. Trans Am Microsc Soc. 1978;97: 171–180.

[64] Adrianov AV, Murakami C, Shirayama Y. Taxonomic study of the Kinorhyncha in Japan. III. *Echinoderes sensibilis* n. sp. (Kinorhyncha: Cyclorhagida) from Tanabe Bay. Zool Stud. 2002;19: 463–473. 10.2108/zsj.19.463 12130824

[65] Sørensen MV, Heiner I, Ziemer O. A new species of *Echinoderes* from Florida (Kinorhyncha, Cyclorhagida). Proc Biol Soc Wash. 2005;118: 499–508.

[66] Sørensen MV. New kinorhynchs from Panama, with a discussion of some phylogenetically significant cuticular structures. Meiofauna Mar. 2006;15: 51–77.

[67] Higgins RP. Indian Ocean Kinorhyncha, 2: Neocentrophyidae, a new homalorhagid family. Proc Biol Soc Wash. 1969;82: 113–128.

[68] Sørensen MV, Pardos F, Herranz M, Rho HS. New data on the genus *Paracentrophyes* (Homalorhagida, Kinorhyncha), with the description of a new species from the West Pacific. Open Zool J. 2010;3: 42–59.

[69] Sørensen MV. A new kinorhynch genus from the Antarctic deep-sea and a new species of *Cephalorhyncha* from Hawaii (Kinorhyncha: Cyclorhagida: Echinoderidae). Org Div Evol.2008; 10.1016/j.ode.2007.11.003

[70] Yamasaki H, Kajihara H, Mawatari SF. First report of kinorhynchs from Hokkaido, including a species of *Pycnophyes* (Pycnophyidae: Homalorhagida). Zootaxa 2012;3425: 23–41.

[71] Sørensen MV, Heiner I, Hansen JG. A comparative morphological study of the kinorhynch genera *Antygomonas* and *Semnoderes* (Kinorhyncha: Cyclorhagida). Helgoland Mar Res. 2009;63: 129–147.

[72] Sørensen MV, Rho HS, Kim D. A new species of the rare genus *Sphenoderes* (Cyclorhagida, Kinorhyncha), with differential notes on *S*. *indicus* Higgins, 1969. Mar Biol Res. 2010;6: 472–484.

[73] Sørensen MV, Rho HS. *Triodontoderes anulap* gen. et sp. nov. – A new cyclorhagid kinorhynch genus and species from Micronesia. JMBA, UK. 2009;89: 1269–1279.

[74] Sørensen MV, Heiner I, Ziemer O, Neuhaus B. *Tubulideres seminoli* gen. et sp. nov. and *Zelinkaderes brightae* sp. nov. (Kinorhyncha, Cyclorhagida) from Florida. Helgoland Mar Res. 2007;61: 247–265.

[75] Sørensen MV, Thormar J. *Wollunquaderes majkenae* gen. et sp. nov.—a new cyclorhagid kinorhynch genus and species from the Coral Sea, Australia. Mar Biodiv. 2010;40: 261–275.

[76] Bauer-Nebelsick M. *Zelinkaderes klepali* sp.n., from shallow water sands of the Red Sea. Ann Nat Mus, Wien 1995;97B: 57–74.

[77] Altenburger A, Rho HS, Chang CY, Sørensen MV. *Zelinkaderes yong* sp. nov. from Korea – the first recording of *Zelinkaderes* (Kinorhyncha: Cyclorhagida) in Asia. Zool Stud. 2015; in press. 10.1186/s40555-014-0103-6 PMC6661445 31966112

[78] Nelles L, Fang BL, Volckaert G, Vandenberghe A, De Wachter R. Nucleotide sequence of a crustacean 18S ribosomal RNA gene and secondary structure of eukaryotic small subunit ribosomal RNAs. Nucleic Acids Res. 1984;12: 8749–8768. 6514572 10.1093/nar/12.23.8749 PMC320417

[79] Yamaguchi S, Endo K. Molecular phylogeny of Ostracoda (Crustacea) inferred from 18S ribosomal RNA sequences: implication for its origin and diversification. Mar Biol. 2003;143: 23–38.

[80] Kim CG, Zhou HZ, Imura Y, Tominaga O, Su Z, Osawa S. Pattern of morphological diversification in the *Leptocarabus* ground beetles (Coleoptera: Carabidae) as deduced from mitochondrial ND5 gene and nuclear 28S rRNA sequences. Mol Biol Evol. 2000; 17: 137–145. 10666713 10.1093/oxfordjournals.molbev.a026226

[81] Luan Y, Mallatt JM, Xie R, Yang Y, Yin W. The phylogenetic positions of three basal-hexapod group (Protura, Diplura, and Collembola) based on ribosomal RNA gene sequences. Mol Biol Evol. 2005;22: 1579–1592. 15845456 10.1093/molbev/msi148

[82] Palumbi SR. Nucleic acids II: The polymerase chain reaction In: Hills DM, Moritz C, Mable BK, editors. Molecular Systematics, second ed Sunderland: Sinauer Associates; 1996 pp. 205–247.

[83] Todaro MA, Kånneby T, Dal Zotto M, Jondelius U. Phylogeny of the Thaumastodermatidae (Gastrotricha: Macrodasyida) inferred from nuclear and mitochondrial sequence data. PLoS One 2011;6: e17892 10.1371/journal.pone.0017892 21455302 PMC3063787

[84] Norén M, Jondelius U. The phylogenetic position of the Prolecithophora (Rhabditophora, ‘Platyhelminthes’). Zool Scr. 2002;31: 403–414.

[85] Norén M, Jondelius U. Phylogeny of the Prolecithophora (Platyhelminthes) inferred from 18S rDNA sequences. Cladistics 1999;15: 103–112. 10.1111/j.1096-0031.1999.tb00252.x 34902908

[86] Giribet G, Carranza S, Baguñà J, Riutort M, Ribera C. First molecular evidence for the existence of a Tardigrada + Arthropoda clade. Mol Bio Evolut. 1996;13: 76–84. 10.1093/oxfordjournals.molbev.a025573 8583909

[87] Whiting MF, Carpenter JC, Wheeler QD, Wheeler WC. The Strepsiptera problem: phylogeny of the holometabolous insect orders inferred from 18S and 28S ribosomal DNA sequences and morphology. Syst Biol. 1997;46: 1–68. 11975347 10.1093/sysbio/46.1.1

[88] Park JK, Ó Foighil D. Sphaeriid and corbiculid clams represent separate heterodont bivalve radiations into freshwater environments. Mol Phylogenet Evolut. 2000;14, 75–88. 10.1006/mpev.1999.0691 10631043

