# Supplementary material for: Phylogeny of Kinorhyncha Based on Morphology and Two Molecular Loci
Source: PLoS One. 2015 Jul 22;10(7):e0133440. doi: 10.1371/journal.pone.0133440 (PMC4511640; doi:10.1371/journal.pone.0133440)
Supplement: S1 File — (DOCX) [file pone.0133440.s001.docx]

**S1 File. Appendix A: The morphological characters**

**Introvert**

1. Size of outer oral styles: 0, outer oral styles of similar size; 1, size of outer oral styles alternate between larger and smaller ones

The character refers to the size of the outer oral styles that in most species are of the same, or nearly the same length (Fig. 4A). The outer oral styles in species of *Dracoderes* (Fig. 4B) and Neocentrophyidae alternate between large well-developed ones and much smaller ones, while outer oral styles are absent in species of *Campyloderes* [46], hence the latter are coded as inapplicable for this character.

1. Composition of outer oral styles: 0, rigid, articulated; 1, soft, non-articulated

Outer orals styles are usually rigid and consist of two or three articulating units (Fig. 4A-B). However, species of Pycnophyidae often have soft, non-articulated outer oral styles (Fig. 4E-F), whereas the outer oral styles of *Condyloderes* are soft and non-articulated as well (Fig. 4D), due to their partial fusion with the mouth cone [47]. Soft and non-articulated outer oral styles are furthermore found in *Triodontoderes anulap* [73].

1. Attachment of outer oral styles and mouth cone: 0, oral styles attach at proximal joint only; 1, inferior part of oral styles partly fused with mouth cone; 2, oral styles completely fused with mouth cone

Whereas most species have free outer oral styles that only attach to the mouth cone proximally (Fig. 4A-B), species of *Condyloderes* have outer oral styles that are partly fused with the mouth cone (Fig. 4D). In species of *Campyloderes* the outer oral styles are either lacking or completely fused with the mouth cone [46] (Fig. 4C).

1. Appearance of primary spinoscalids: 0, single; 1, bifurcated

The character refers to a special condition known only from species of *Cateria* [59] and New Genus (Fig. 4G). Whereas the primary spinoscalids are regular, single appendages in the first introvert ring, the primary spinoscalids of *Cateria* and New Genus are basally bifurcated, so it appears that the ring has twenty instead of ten scalids. A similar condition is most probably present in *Franciscideres kalenesos* [19], but this needs to be confirmed, hence for now this taxon is coded as uncertain.

1. Number of trichoscalids: 0, six trichoscalids; 1, seven trichoscalids, 2, nine trichoscalids; 3, fourteen trichoscalids

The character refers to the number of trichoscalids (Fig. 4H) that appear posterior to the last spinoscalid ring of the introvert. Trichoscalids are apparently present in all kinorhynch species, but the exact number is still uncertain for some taxa.

1. Attachment of trichoscalids: 0, trichoscalids attach directly on introvert; 1, trichoscalids attach on trichoscalid plates

In a majority of kinorhynchs, the trichoscalids attach directly on the cuticle in the most posterior part of the introvert. However, in all species of Echinoderidae, the trichoscalids attach to large plates, trichoscalid plates (Fig. 4H), that are connected posteriorly to the placids of the neck region.

**Neck**

1. Neck region forms segment-like ring: 0, absent; 1, present

Nearly all known kinorhynch species have a neck region, formed by small, rectangular plates, called placids [3] (Fig. 5B-E). However, the neck in *Franciscideres kalenesos* [19] and New Genus (Fig. 5A) is completely different, as it here forms a large, segment-like ring.

1. Placids in neck: 0, absent; 1, present

The character refers to the presence of placids (Fig. 5B-E) in the neck region. The placids serve as a closing mechanism when the head is retracted into the trunk (Fig. 5B-D). The character differs from character 7 by the coding of *Cateria gerlachi*, that on one hand does not have clearly differentiated placids (see Fig. 5.1.9B in Neuhaus 2013 [3]), but on the other hand also lacks the large segment-like neck as found in *F. kalenesos* and New Genus (Fig. 5A).

1. Number of placids in neck: 0, six; 1, seven; 2, eight; 3, nine; 4, fourteen, 5, sixteen

The number of placids in the neck region varies between the kinorhynch genera and families, and may go from only six to nine placids, as found in species of Allomalorhagida (Fig. 5C) and up to fourteen or sixteen placids, as present in many cyclorhagids (Fig. 5B). Taxa without placids are coded as inapplicable for this character.

1. Ornamentation of placids: 0, placids smooth; 1, placids with conspicuous knobby projections

Whereas most species have placids with a regular smooth cuticular surface (Fig. 5B-C), species of *Condyloderes* are recognized by the conspicuous knobby projections in their placids (Fig. 5D). Taxa without placids are coded as inapplicable for this character.

1. Shape of distal margins of placids: 0, placids rectangular with straight margin and angular corners; 1, placids elongate with distal tripartition

Most species have regular angular anterior corners on their placids, but some species of *Zelinkaderes* and *Triodontoderes anulap* have distally tripartite placids (Fig. 5E), and this has been suggested as synapomorphy for species of the two genera [73]. It was however not possible to determine whether this tripartition was present in the recently described species *Zelinkaderes yong* [77], and likewise the condition of the character could not be examined in *Zelinkaderes* sp. 1, hence these taxa are coded as uncertain for this character. Taxa without placids are coded as inapplicable for this character.

1. Attachment of placids: 0, placids fused with first trunk segment; 1, placids attach through joint

In some species the placids are apparently direct cuticular extensions from segment 1 (Fig. 5E), whereas other species have a distinct articulation between their placids and segment 1 (Fig. 5B-D). Taxa without placids are coded as inapplicable for this character.

1. Symmetry of closing apparatus (excl. midventral placid): 0, radial symmetrical; 1, bilateral symmetrical

In most species of Allomalorhagida the placids form a bilateral symmetrical closing apparatus (Fig. 5C), whereas most species of Cyclorhagida show a radial symmetrical placid arrangement (Fig. 5B). The exceptions among cyclorhagids are species of *Semnoderes* and *Sphenoderes* that have a special bilateral symmetrical clamshell-like closing apparatus.

**Trunk**

1. Anterior margin of first trunk segment with lateral projections: 0, absent; 1, present

The character refers to the anterolateral corners in species of Pycnophyidae and Neocentrophyidae that often extend into horn-like projections (Fig. 6A-C).

1. Composition of first segment: 0, complete ring; 1, one tergal and one narrow, midventral plate; 2, one tergal and one broad sternal plate, eventually partially or fully differentiated into mid- and episternal plates.

The character refers to the composition of segment 1, and character state 2 is linked to the following character, character 16. As described under character 16, it is assumed that the sternal plate in species of Pycnophyidae and Neocentrophyidae went through a transitional differentiation of their sternal plates, into two episternal and one midsternal plate. Hence, all species with either a single, broad midsternal plate, i.e., as present in species of *Neocentrophyes* and *Mixtophyes* (Fig. 6A), species with partly differentiated episternal and midsternal as observed in *Paracentrophyes* (Fig. 6B), or species with fully differentiated episternal and midsternal plate, i.e., *Pycnophyes* (Fig. 6C) and *Kinorhynchus* code for character state 2. Character state 1, the possessing of a tergal plate and a rather narrow midventral plate, is present in *Triodontoderes anulap* [73] and *Cateria gerlachi* [59] only, whereas state 0, segment 1 consisting of a closed ring is present in all remaining taxa.

1. Differentiation of sternal plate in taxa with one tergal and broad sternal plate: 0, sternal plate undifferentiated; 1, sternal plate partially subdivided; 2, sternal plate differentiated into episternal and midsternal plates [ordered]

The character applies to all taxa that are coded as state 2 in character 15. The character is based on the assumption that the ventral side of segment 1 in species of Pycnophyidae and Neocentrophyidae either went through a transitional differentiation series going from having a single, broad sternal plate (Fig. 6A) and towards having the ventral side differentiated into two episternal and one midsternal plate (Fig. 6C), or oppositely, from differentiated midsternal and episternal plates towards having a single plate. It is assumed that the condition with partly differentiated midsternal and episternal plates (state 1), as present in species of *Paracentrophyes* (Fig. 6B), represents an intermediate transitional stage between having a single sternal plate and having fully differentiated midsternal and episternal plates (state 2). Hence, the character is treated as symmetrically ordered. All taxa that were not coded as state 2 in character 15 are coded as inapplicable for this character.

1. Appearance of anterior margin of first segment: 0, straight; 1, with middorsal and midventral sinuate incisions; 2, with deep middorsal and midventral incisions [ordered]

The character refers to the shape of the anterior margin of segment 1 in species of *Antygomonas*, *Semnoderes* and *Sphenoderes*. In most kinorhynch species, the anterior margin of segment 1 is completely straight (see, e.g., Fig. 5B and Fig. 7F). However, in species of *Antygomonas* (Fig. 7A) the anterior margin of segment 1 shows broadly sinuate middorsal and midventral incisions, and in species of *Semnoderes* (Fig. 7B) and *Sphenoderes* (Fig. 7C) the segment margins have deep wedge-shaped incisions. Studies [71], [72] have suggested that the modifications of segment 1 in these three genera are part of a transformation series, with the condition in *Antygomonas* being an intermediate stage between the straight segment margin and the rather extremely wedge-shaped incision in species of *Semnoderes* and *Sphenoderes*. Hence, the character is treated as symmetrically ordered.

1. Composition of second segment: 0, complete ring, or with one tergal plate and partial division into two sternal plates, or complete division into one tergal and two sternal plates; 1, one tergal and one sternal plate

The character refers to the composition of segment 2, and character state 0 is linked to the following character, character 19. The genera of Echinoderidae are distinguished by the composition of segment 2 that may either form a closed ring as in species of *Echinoderes* (Fig. 6D), be differentiated into one tergal and two sternal plates as in species of *Fissuroderes* and *Polacanthoderes* (Fig. 6F-G), or represent intermediate stages in between these conditions, as found in species of *Cephalorhyncha* and *Meristoderes* (Fig. 18E). Character state 0 refers to all taxa that have segment 2 as a closed ring, with differentiated tergal and sternal plates, or displaying an intermediate stage in this transition. Character state 1, i.e., having segment 2 consisting of a tergal and a single sternal plate, is present in species of *Cateria* only [59].

1. Transition through formation of sternal plates in segment 2: 0, sternal plates not developed; 1, lateral fissures partially developed, no midsternal fissure; 2, lateral fissures fully developed, midsternal fissure partially developed; 3, sternal plates fully differentiated [ordered]

The character applies to all taxa that are coded as state 0 in character 18. The character is based on the assumption that segment 2 in species of Echinoderidae went through a transitional series from either being composed of a closed cuticular ring towards being differentiated in to a segment composed of a tergal and two sternal plates, or oppositely, being composed of differentiated plates that fused into a closed ring. A second segment composed of a closed ring (state 0) is present in all species of *Echinoderes* (Fig. 6D) and is diagnostic for this genus, e.g. [3] and [37], whereas a second segment composed of completely differentiated tergal and sternal plates (state 3) is present in species of *Polacanthoderes* (Fig. 6G) and *Fissuroderes* (Fig. 6F), see also [34], [42], [69]. An intermediate stage in this transition, being close to having a closed cuticular ring, is present in species of *Meristoderes* (Fig. 6E) that have partially developed lateral fissures (state 1), but no traces of the midsternal fissure [35], [36], [42]. One additional intermediate stage with fully developed lateral fissures but only partially developed midsternal fissure (state 2) is present in species of *Cephalorhyncha* [17], [34], [69]. We consider this stage to be closer to the condition with fully differentiated plates. The direction of this transformation series remains uncertain, hence, the character is treated as symmetrically ordered.

1. Third and fourth segment: 0, one tergal plate with midventral articulation; 1, one tergal and one sternal plate; 2, one tergal and two sternal plates; 3, segments are closed rings

The character refers to the composition of segments 3 and 4. A great majority of all taxa have segments composed of a tergal and two sternal plates (state 2). Exceptions are species of *Franciscideres* and *Zelinkaderes* that have segments composed of a single tergal plate with midventral articulation (state 0), species of *Cateria* where these segments, like their two anterior ones, are composed of a tergal and a single sternal plate (state 1), and New Genus that has segments 3 and 4 composed of closed cuticular rings (state 3).

1. Seventh to tenth segment: 0, one tergal plate with midventral articulation; 1, one tergal and two sternal plates; 2, segments are closed rings

The character refers to the composition of segments 7 to 10 that may differ from the more anterior segments. Again, a large majority of all taxa have segments composed of a tergal and two sternal plates (state 1). Exceptions are species of *Cateria*, *Franciscideres*, *Triodontoderes* and *Zelinkaderes* that have segments composed of a single tergal plate with midventral articulation (state 0), and New Genus that has segments 7 to 10 composed of closed cuticular rings (state 2).

1. Terminal segment: 0, one tergal and two sternal plates; 1, one tergal and one sternal plate; 2, single tergal plate with midventral joint; 3, closed ring; 4, two tergal and two sternal plates

The terminal trunk segment consists in most taxa of a tergal and two sternal plates (state 0). However, the composition of this segment appears to show more variability than the other trunk segments. For instance, it may also be composed of single tergal and sternal plates, paired tergal and sternal plates, or just a single closed or ventrally articulating plate. The character even shows variability within some genera, e.g., *Fissuroderes* [34] and *Echinoderes* [41] and its phylogenetic significance can be questioned. However, we include the character to test if it contains some information.

1. Segment 1 extended into spinose midventral process: 0, absent; 1 present

The character refers to the presence of a special spinose process that may extend from a midventral position at the posterior margin of segment 1 (Fig. 7D). The character is considered genus diagnostic for species of *Centroderes*, and the trait is not found outside this genus [48], [49].

1. Trunk segments with lateroventral notches in posterior margins: 0, absent; 1 present

The character refers to various genera, typically with rather thin body cuticle such as in *Antygomonas* and *Zelinkaderes*, that show deep lateroventral notches in the posterior segment margins (Fig. 7E). Lateroventral spines may appear in the notches, but this is not necessary the case.

**Trunk appendages**

1. Trunk with non-articulated, middorsal structures at the posterior segment margins: 0, absent; 1 present

The character refers to the presence of non-articulated middorsal structures, i.e., direct extensions of the segmental cuticle (Fig. 7G-H). The character should not be confused with acicular spines (e.g. Fig. 7I) that attach to the trunk through articulations. Middorsal specializations are typically found in species of Pycnophyidae (Fig. 7G) and Neocentrophyidae (Fig. 7H).

1. Middorsal specializations extended into spinose processes: 0, protruding well beyond posterior segment margin; 1, not or only slightly protruding beyond posterior segment margin

When comparing the length of non-articulated middorsal structures, in particular on the more posterior trunk segments, it becomes clear that the processes in species of Pycnophyidae are very short and hardly projecting (if projecting at all) beyond the posterior segment margin (state 1) (Fig. 7G). Oppositely, in species of Neocentrophyidae, the structures are often conspicuously larger and spinose, and project well beyond the posterior segment margin (state 0) (Fig. 7H). The character is linked to character 26, and all taxa without non-articulated, middorsal structures are coded as inapplicable for this character.

1. Trunk with setae: 0, absent; 1, present

A common trait for all species of Pycnophyidae and Neocentrophyidae is the presence of minute setae (Fig. 7H). Such setae are not present in any other taxa outside these families.

1. Overall distribution of middorsal spines: 0, spines can occur on any segment from segment 1 to 11; 1, spines restricted to segments between segment 4 and 8

The spine distribution across the kinorhynch species is obviously homoplastic to some extent. However, certain trends can be identified. For instance, representatives of many families have the ability to develop middorsal spines on all trunk segments (state 0), whereas middorsal spines in species of Echinoderidae apparently always are restricted to segments 4 to 8 (state 1). The middorsal spines in species of Echinoderidae do not necessarily appear on all segments from 4 to 8, and may for instance be restricted to segments 4, 6 and 8, only segment 4, or not be present at all, but reports of middorsal spines occurring on any other segment between 4 and 8 are extremely rare. The character is made to include the information that formation of middorsal spines on other segments than 4 to 8 seems to be genetically suppressed in species of Echinoderidae. Species of Pycnophyidae and Neocentrophyidae that apparently are unable to form spines at all on segments 1 to 9 (see character 29) are coded as inapplicable for this character.

1. Spines restricted to segments 10 and 11: 0, absent; 1, present

Whereas many, especially cyclorhagid, kinorhynchs have spines in various positions on most segments, species of Neocentrophyidae and Pycnophyidae are incapable of forming any spines at all on segment 1 to 9. If spines occur among these species, they are restricted to segments 10 and 11.

1. Arrangement of dorsal spines: 0, aligned middorsally; 1, alternatingly laterally displaced

In basically all species with dorsal spines, the spines will be arranged along the middorsal line. However, in species of *Dracoderes* the dorsal spines are alternatingly displaced to the lateral sides (Fig. 7I). This character trait is diagnostic for the genus [31]. Species of Pycnophyidae and Neocentrophyidae are coded as inapplicable for this character.

1. Overall distribution of lateroventral spines: 0, spines can occur on any segment from segment 1 to 10; 1, spines restricted to segments between segment 6 and 9

As it is the case with middorsal spines (see character 28), lateroventral spines may in some genera occur on all or most segments (state 0), whereas they in species of Echinoderidae apparently are restricted to certain segments, i.e., segments 6 to 9 (state 1). Lateroventral spines in species of Echinoderidae do not necessarily appear on all segments from 6 to 9, but apparently they occur extremely rarely on any other segments. The character is made to include the information that formation of lateroventral spines on other segments than 6 to 9 seems to be genetically suppressed in species of Echinoderidae. Species of Pycnophyidae and Neocentrophyidae are coded as inapplicable for this character.

1. Segment 1 with a pair of extraordinary long lateroventral spines situated next to pair of very short ventrolateral spines: 0, absent; 1, present

State 1 in this character refers to a special condition found in species of *Campyloderes* only [45], [46] (Fig. 7F).

1. Segment 1 with a pair of extraordinary long ventrolateral spines: 0, absent; 1, present

State 1 in this character refers to a special condition found in species of *Centroderes* only [48], [49] (Fig. 7D).

1. Cuspidate spines: 0, absent; 1, present

The character refers to the presence of a special kind of spines, so-called cuspidate spines, that are found in various genera of Cyclorhagida. Cuspidate spines have a peculiar bottle- or pin-shape, and attach in the thick end (Fig. 7E).

1. Sexual dimorphism expressed as presence of ventromedial tubes on segment 2 in males: 0, absent; 1, present

The character refers to a sexually dimorphic character found in most, but not all, species of Pycnophyidae. In nearly all species of this family, males would have a pair of rather thick ventromedial tubes on segment 2 (Fig. 7J). The tubes are present in all Pycnophyidae species included in the present data matrix.

1. Males with crenulated spines on segment 10: 0, absent; 1, present

The character refers to a sexually dimorphic character found in various genera of Cyclorhagida. The character is expressed on the shape of the middorsal and laterodorsal/midlateral spines of segment 10. Whereas females typically would have regular acicular spines in these positions (or alternatively no spines at all), males of some species show so-called crenulated spines, i.e., a distally annulated and flexible kind of spines (Fig. 7K).

1. Males with penile spines: 0, absent; 1, present

The character refers to a sexually dimorphic character found in various kinorhynch groups, including Echinoderidae, Dracoderidae, Pycnophyidae and Neocentrophyidae. Penile spines appear in males only, and appear in a midlateral position from the intersegmental transition between segment 10 and 11 (Fig. 7L). Penile spines may be either long, thin and flexible tubes, or much stouter, triangular protuberances. Both kinds of penile spines may occur in the same species.

**Terminal spines**

1. Lateral terminal accessory spines in one or both sexes: 0, absent; 1 present

The character refers to the presence of lateral terminal accessory spines, without taking any eventual sexually dimorphic differences into account. Lateral terminal accessory spines (Fig. 6H) are located on segment 11 in a lateral accessory position, i.e., slightly more dorsal than the lateral terminal spines.

1. Gender determined presence of LTAS: 0, present in both sexes; 1, present in females only

In most (but not all) species of Echinoderidae, lateral terminal accessory spines are present in females only. Taxa without lateral terminal accessory spines are coded as inapplicable for this character.

1. Lateral terminal spines: 0, absent; 1 present

The character refers to the presence of lateral terminal spines (Fig. 6H), which are located on segment 11 in a lateroventral positions, i.e., slightly more ventral than eventual lateral terminal accessory spines.

1. Midterminal spine: 0, absent; 1 present

The character refers to the presence of a midterminal spine (Fig. 6H), i.e., a strong spine that articulates with the most caudal point of the trunk.

1. Midterminal process: 0, absent; 1 present

The character refers to the presence of a midterminal process (Fig. 6I), i.e., a process that appears as a direct, posterior extension from the most caudal point of the trunk. Among the species of the present analysis, a midterminal process is only present in *Paracentrophyes quadridentatus* [68] and species of *Neocentrophyes* [5].
