# Supplementary material for: Phylogeny of Kinorhyncha Based on Morphology and Two Molecular Loci
Source: PLoS One. 2015 Jul 22;10(7):e0133440. doi: 10.1371/journal.pone.0133440 (PMC4511640; doi:10.1371/journal.pone.0133440)
Supplement: S2 File — (DOCX) [file pone.0133440.s002.docx]

**S2 File. Appendix B: The morphological matrix**

| **Genus and species** | **1** | **2** | **3** | **4** | **5** | **6** | **7** | **8** | **9** | **10** | **11** | **12** | **13** | **14** | **15** | **16^*^** | **17^*^** | **18** | **19^*^** | **20** | **21** |
| --- | --- | --- | --- | --- | --- | --- | --- | --- | --- | --- | --- | --- | --- | --- | --- | --- | --- | --- | --- | --- | --- |
| *Antygomonas caeciliae* | 0 | 0 | 0 | 0 | 3 | 0 | 0 | 1 | 5 | 0 | 0 | 1 | 1 | 0 | 0 | - | 1 | 0 | 3 | 2 | 1 |
| *Antygomonas paulae* | 0 | 0 | 0 | 0 | 3 | 0 | 0 | 1 | 5 | 0 | 0 | 1 | 1 | 0 | 0 | - | 1 | 0 | 3 | 2 | 1 |
| *Antygomonas* sp. 2 | 0 | 0 | 0 | 0 | ? | 0 | 0 | 1 | 5 | 0 | 0 | 1 | 1 | 0 | 0 | - | 1 | 0 | 3 | 2 | 1 |
| *Antygomonas* sp. 3 | 0 | 0 | 0 | 0 | ? | 0 | 0 | 1 | 5 | 0 | 0 | 1 | 1 | 0 | 0 | - | 1 | 0 | 3 | 2 | 1 |
| *Campyloderes* cf. *vanhoeffeni* | - | - | 2 | 0 | 3 | 1 | 0 | 1 | 4 | 0 | 0 | 1 | 0 | 0 | 0 | - | 0 | 0 | 3 | 2 | 1 |
| *Campyloderes* sp. 1 | - | - | 2 | 0 | ? | ? | 0 | 1 | 4 | 0 | 0 | 1 | 0 | 0 | 0 | - | 0 | 0 | 3 | 2 | 1 |
| *Campyloderes* sp. 2 | - | - | 2 | 0 | ? | ? | 0 | 1 | 4 | 0 | 0 | 1 | 0 | 0 | 0 | - | 0 | 0 | 3 | 2 | 1 |
| *Cateria gerlachi* | 0 | 0 | 0 | 1 | 3 | 0 | 0 | 0 | - | - | - | - | 0 | 0 | 1 | - | 0 | 1 | 3 | 1 | 0 |
| *Centroderes spinosus* | 0 | 0 | 0 | 0 | 3 | 0 | 0 | 1 | 5 | 0 | 0 | 1 | 0 | 0 | 0 | - | 0 | 0 | 3 | 2 | 1 |
| *Centroderes* sp. | 0 | 0 | 0 | 0 | 3 | 0 | 0 | 1 | 5 | 0 | 0 | 1 | 0 | 0 | 0 | - | 0 | 0 | 3 | 2 | 1 |
| *Cephalorhyncha* sp. 1 | 0 | 0 | 0 | 0 | 0 | 1 | 0 | 1 | 5 | 0 | 0 | 1 | 0 | 0 | 0 | - | 0 | 0 | 2 | 2 | 1 |
| *Condyloderes* sp. 1 | 0 | 1 | 1 | 0 | 3 | 0 | 0 | 1 | 5 | 1 | 0 | 1 | 0 | 0 | 0 | - | 0 | 0 | 3 | 2 | 1 |
| *Condyloderes* sp. 2 | ? | ? | ? | 0 | 3 | 0 | 0 | 1 | ? | 1 | 0 | 1 | 0 | 0 | 0 | - | 0 | 0 | 3 | 2 | 1 |
| *Dracoderes abei* | 1 | 0 | 0 | 0 | 2 | 0 | 0 | 1 | 3 | 0 | 0 | 1 | 1 | 0 | 0 | - | 0 | 0 | 3 | 2 | 1 |
| *Dracoderes nidhug* | 1 | 0 | 0 | 0 | 2 | 0 | 0 | 1 | 3 | 0 | 0 | 1 | 1 | 0 | 0 | - | 0 | 0 | 3 | 2 | 1 |
| *Echinoderes ajax* | 0 | 0 | 0 | 0 | 0 | 1 | 0 | 1 | 5 | 0 | 0 | 1 | 0 | 0 | 0 | - | 0 | 0 | 0 | 2 | 1 |
| *Echinoderes astridae* | 0 | 0 | 0 | 0 | 0 | 1 | 0 | 1 | 5 | 0 | 0 | 1 | 0 | 0 | 0 | - | 0 | 0 | 0 | 2 | 1 |
| *Echinoderes aureus* | 0 | 0 | 0 | 0 | 0 | 1 | 0 | 1 | 5 | 0 | 0 | 1 | 0 | 0 | 0 | - | 0 | 0 | 0 | 2 | 1 |
| *Echinoderes capitatus* | 0 | 0 | 0 | 0 | 0 | 1 | 0 | 1 | 5 | 0 | 0 | 1 | 0 | 0 | 0 | - | 0 | 0 | 0 | 2 | 1 |
| *Echinoderes dujardinii* | 0 | 0 | 0 | 0 | 0 | 1 | 0 | 1 | 5 | 0 | 0 | 1 | 0 | 0 | 0 | - | 0 | 0 | 0 | 2 | 1 |
| *Echinoderes gerardi* | 0 | 0 | 0 | 0 | 0 | 1 | 0 | 1 | 5 | 0 | 0 | 1 | 0 | 0 | 0 | - | 0 | 0 | 0 | 2 | 1 |
| *Echinoderes horni* | 0 | 0 | 0 | 0 | 0 | 1 | 0 | 1 | 5 | 0 | 0 | 1 | 0 | 0 | 0 | - | 0 | 0 | 0 | 2 | 1 |
| *Echinoderes marthae* | 0 | 0 | 0 | 0 | 0 | 1 | 0 | 1 | 5 | 0 | 0 | 1 | 0 | 0 | 0 | - | 0 | 0 | 0 | 2 | 1 |
| *Echinoderes microaperturus* | 0 | 0 | 0 | 0 | 0 | 1 | 0 | 1 | 5 | 0 | 0 | 1 | 0 | 0 | 0 | - | 0 | 0 | 0 | 2 | 1 |
| *Echinoderes sensibilis* | 0 | 0 | 0 | 0 | 0 | 1 | 0 | 1 | 5 | 0 | 0 | 1 | 0 | 0 | 0 | - | 0 | 0 | 0 | 2 | 1 |
| *Echinoderes setiger* | ? | ? | ? | ? | ? | ? | 0 | 1 | 5 | 0 | 0 | 1 | 0 | 0 | 0 | - | 0 | 0 | 0 | 2 | 1 |
| *Echinoderes spinifurca* | 0 | 0 | 0 | 0 | 0 | 1 | 0 | 1 | 5 | 0 | 0 | 1 | 0 | 0 | 0 | - | 0 | 0 | 0 | 2 | 1 |
| *Echinoderes truncatus* | 0 | 0 | 0 | 0 | 0 | 1 | 0 | 1 | 5 | 0 | 0 | 1 | 0 | 0 | 0 | - | 0 | 0 | 0 | 2 | 1 |
| *Echinoderes* sp. 1 | 0 | 0 | 0 | 0 | 0 | 1 | 0 | 1 | 5 | 0 | 0 | 1 | 0 | 0 | 0 | - | 0 | 0 | 0 | 2 | 1 |
| *Fissuroderes sorenseni* | 0 | 0 | 0 | 0 | 0 | 1 | 0 | 1 | 5 | 0 | 0 | 1 | 0 | 0 | 0 | - | 0 | 0 | 3 | 2 | 1 |
| *Fissuroderes thermoi* | 0 | 0 | 0 | ? | 0 | 1 | 0 | 1 | 5 | 0 | 0 | 1 | 0 | 0 | 0 | - | 0 | 0 | 3 | 2 | 1 |
| *Franciscideres kalenesos* | 0 | 0 | 0 | ? | ? | 0 | 1 | 0 | - | - | - | - | 0 | 0 | 0 | - | 0 | 0 | 0 | 0 | 0 |
| *Kinorhynchus giganteus* | ? | ? | ? | ? | ? | ? | 0 | 1 | 2 | 0 | 0 | 1 | 1 | 1 | 2 | 2 | 0 | 0 | 3 | 2 | 1 |
| *Kinorhynchs yushini* | 0 | 1 | 0 | 0 | 3 | 0 | 0 | 1 | 0 | 0 | 0 | 1 | 1 | 1 | 2 | 2 | 0 | 0 | 3 | 2 | 1 |
| *Meristoderes macracanthus* | 0 | 0 | 0 | 0 | 0 | 1 | 0 | 1 | 5 | 0 | 0 | 1 | 0 | 0 | 0 | - | 0 | 0 | 1 | 2 | 1 |
| *Meristoderes* sp. | 0 | 0 | 0 | 0 | 0 | 1 | 0 | 1 | 5 | 0 | 0 | 1 | 0 | 0 | 0 | - | 0 | 0 | 1 | 2 | 1 |
| *Mixtophyes abyssalis* | 1 | 0 | 0 | 0 | ? | 0 | 0 | 1 | 1 | 0 | 0 | 1 | 1 | 1 | 2 | 0 | 0 | 0 | 3 | 2 | 1 |
| *Neocentrophyes intermedius* | 1 | 0 | 0 | 0 | 3 | 0 | 0 | 1 | 1 | 0 | 0 | 1 | 1 | 1 | 2 | 0 | 0 | 0 | 3 | 2 | 1 |
| *Neocentrophyes satyai* | 1 | 0 | 0 | 0 | 3 | 0 | 0 | 1 | 1 | 0 | 0 | 1 | 1 | 1 | 2 | 0 | 0 | 0 | 3 | 2 | 1 |
| *New Genus* | 0 | 0 | 0 | 1 | ? | 0 | 1 | 0 | - | - | - | - | 0 | 0 | 0 | - | 0 | 0 | 0 | 3 | 2 |
| *Paracentrophyes anurus* | 1 | 0 | 0 | 0 | 3 | 0 | 0 | 1 | 1 | 0 | 0 | 1 | 1 | 1 | 2 | 1 | 0 | 0 | 3 | 2 | 1 |
| *Paracentrophyes quadridentatus* | 1 | 0 | 0 | 0 | 3 | 0 | 0 | 1 | 1 | 0 | 0 | 1 | 1 | 1 | 2 | 1 | 0 | 0 | 3 | 2 | 1 |
| *Polacanthoderes martinezi* | 0 | 0 | 0 | 0 | 0 | 1 | 0 | 1 | 5 | 0 | 0 | 1 | 0 | 0 | 0 | - | 0 | 0 | 3 | 2 | 1 |
| *Pycnophyes communis* | ? | ? | ? | ? | ? | ? | 0 | 1 | 0 | 0 | 0 | 1 | 1 | 1 | 2 | 2 | 0 | 0 | 3 | 2 | 1 |
| *Pycnophyes dentatus* | ? | ? | ? | 0 | 3 | 0 | 0 | 1 | 0 | 0 | 0 | 1 | 1 | 1 | 2 | 2 | 0 | 0 | 3 | 2 | 1 |
| *Pycnophyes greenlandicus* | 0 | 1 | 0 | 0 | 3 | 0 | 0 | 1 | 0 | 0 | 0 | 1 | 1 | 1 | 2 | 2 | 0 | 0 | 3 | 2 | 1 |
| *Pycnophyes kielensis* | 0 | 1 | 0 | 0 | 3 | 0 | 0 | 1 | 2 | 0 | 0 | 1 | 1 | 1 | 2 | 2 | 0 | 0 | 3 | 2 | 1 |
| *Pycnophyes oshoroensis* | ? | ? | ? | ? | ? | ? | 0 | 1 | 0 | 0 | 0 | 1 | 1 | 1 | 2 | 2 | 0 | 0 | 3 | 2 | 1 |
| *Pycnophyes robustus* | ? | ? | ? | ? | ? | ? | 0 | 1 | 0 | 0 | 0 | 1 | 1 | 1 | 2 | 2 | 0 | 0 | 3 | 2 | 1 |
| *Pycnophyes tubuliferus* | 0 | 1 | 0 | 0 | 3 | 0 | 0 | 1 | 0 | 0 | 0 | 1 | 1 | 1 | 2 | 2 | 0 | 0 | 3 | 2 | 1 |
| *Pycnophyes zelinkaei* | 0 | 1 | 0 | 0 | 3 | 0 | 0 | 1 | 0 | 0 | 0 | 1 | 1 | 1 | 2 | 2 | 0 | 0 | 3 | 2 | 1 |
| *Semnoderes armiger* (Italy) | 0 | 0 | 0 | 0 | 3 | 0 | 0 | 1 | 5 | 0 | 0 | 1 | 1 | 0 | 0 | - | 2 | 0 | 3 | 2 | 1 |
| *Semnoderes armiger* (Norway) | 0 | 0 | 0 | 0 | 3 | 0 | 0 | 1 | 5 | 0 | 0 | 1 | 1 | 0 | 0 | - | 2 | 0 | 3 | 2 | 1 |
| *Sphenoderes poseidon* | 0 | 0 | 0 | ? | ? | 0 | 0 | 1 | 5 | 0 | 0 | 1 | 1 | 0 | 0 | - | 2 | 0 | 3 | 2 | 1 |
| *Triodontoderes anulap* | 0 | 1 | 0 | 0 | 3 | 0 | 0 | 1 | 4 | 0 | 1 | 0 | 0 | 0 | 1 | - | 0 | 0 | 3 | 2 | 0 |
| *Tubulideres seminoli* | 0 | 0 | 0 | 0 | 3 | 0 | 0 | 1 | 5 | 0 | 0 | 1 | 0 | 0 | 0 | - | 0 | 0 | 3 | 2 | 1 |
| *Wollunquaderes majkenae* | 0 | 0 | 0 | 0 | 3 | 0 | 0 | 1 | 5 | 0 | 0 | 1 | 0 | 0 | 0 | - | 0 | 0 | 3 | 2 | 1 |
| *Zelinkaderes brightae* | 0 | 0 | 0 | 0 | 3 | 0 | 0 | 1 | 5 | 0 | 1 | 0 | 0 | 0 | 0 | - | 0 | 0 | 0 | 0 | 0 |
| *Zelinkaderes klepali* | 0 | 0 | 0 | 0 | 3 | 0 | 0 | 1 | 5 | 0 | 1 | 0 | 0 | 0 | 0 | - | 0 | 0 | 0 | 0 | 0 |
| *Zelinkaderes yong* | 0 | 0 | 0 | 0 | 3 | 0 | ? | ? | ? | ? | ? | 0 | 0 | 0 | 0 | - | 0 | 0 | 0 | 0 | 0 |
| *Zelinkaderes* sp. 1 | 0 | 0 | 0 | 0 | ? | ? | ? | ? | ? | ? | ? | 0 | 0 | 0 | 0 | - | 0 | 0 | 0 | 0 | 0 |

| **Genus and species** | **22** | **23** | **24** | **25** | **26** | **27** | **28** | **29** | **30** | **31** | **32** | **33** | **34** | **35** | **36** | **37** | **38** | **39** | **40** | **41** | **42** |
| --- | --- | --- | --- | --- | --- | --- | --- | --- | --- | --- | --- | --- | --- | --- | --- | --- | --- | --- | --- | --- | --- |
| *Antygomonas caeciliae* | 0 | 0 | 1 | 0 | - | 0 | 0 | 0 | 0 | 0 | 0 | 0 | 1 | 0 | 0 | 0 | 1 | 0 | 1 | 1 | 0 |
| *Antygomonas paulae* | 0 | 0 | 1 | 0 | - | 0 | 0 | 0 | 0 | 0 | 0 | 0 | 1 | 0 | 0 | 0 | 1 | 0 | 1 | 1 | 0 |
| *Antygomonas* sp. 2 | 0 | 0 | 1 | 0 | - | 0 | 0 | 0 | 0 | 0 | 0 | 0 | 1 | 0 | 0 | 0 | 1 | 0 | 1 | 1 | 0 |
| *Antygomonas* sp. 3 | 0 | 0 | 1 | 0 | - | 0 | 0 | 0 | 0 | 0 | 0 | 0 | 1 | 0 | 0 | 0 | 1 | 0 | 1 | 1 | 0 |
| *Campyloderes* cf. *vanhoeffeni* | 0 | 0 | 0 | 0 | - | 0 | 0 | 0 | 0 | 0 | 1 | 0 | 0 | 0 | 0 | 0 | 1 | 0 | 1 | 1 | 0 |
| *Campyloderes* sp. 1 | 0 | 0 | 0 | 0 | - | 0 | 0 | 0 | 0 | 0 | 1 | 0 | 0 | 0 | ? | ? | 1 | 0 | 1 | 1 | 0 |
| *Campyloderes* sp. 2 | 0 | 0 | 0 | 0 | - | 0 | 0 | 0 | 0 | 0 | 1 | 0 | 0 | 0 | ? | ? | 1 | 0 | 1 | 1 | 0 |
| *Cateria gerlachi* | 2 | 0 | 1 | 0 | - | 0 | 0 | 0 | 0 | 0 | 0 | 0 | 0 | 0 | 0 | 0 | 1 | 0 | 1 | 1 | 0 |
| *Centroderes spinosus* | 0 | 1 | 0 | 0 | - | 0 | 0 | 0 | 0 | 0 | 0 | 1 | 0 | 0 | 1 | 0 | 1 | 0 | 1 | 1 | 0 |
| *Centroderes* sp. | 0 | 1 | 0 | 0 | - | 0 | 0 | 0 | 0 | 0 | 0 | 1 | 0 | 0 | 1 | 0 | 1 | 0 | 1 | 1 | 0 |
| *Cephalorhyncha* sp. 1 | ? | 0 | 0 | 0 | - | 0 | 1 | 0 | 0 | 1 | 0 | 0 | 0 | 0 | 0 | 1 | 1 | 1 | 1 | 0 | 0 |
| *Condyloderes* sp. 1 | 1 | 0 | 0 | 0 | - | 0 | 0 | 0 | 0 | 0 | 0 | 0 | 1 | 0 | 0 | 0 | 0 | - | 1 | 1 | 0 |
| *Condyloderes* sp. 2 | ? | 0 | 0 | 0 | - | 0 | 0 | 0 | 0 | 0 | 0 | 0 | 1 | 0 | ? | ? | ? | ? | 1 | 1 | 0 |
| *Dracoderes abei* | 0 | 0 | 0 | 0 | - | 0 | 0 | 0 | 1 | 1 | 0 | 0 | 0 | 0 | 0 | 1 | 0 | - | 1 | 0 | 0 |
| *Dracoderes nidhug* | 0 | 0 | 0 | 0 | - | 0 | 0 | 0 | 1 | 1 | 0 | 0 | 0 | 0 | 0 | 1 | 0 | - | 1 | 0 | 0 |
| *Echinoderes ajax* | 0 | 0 | 0 | 0 | - | 0 | 1 | 0 | 0 | 1 | 0 | 0 | 0 | 0 | 0 | 1 | 1 | 1 | 1 | 0 | 0 |
| *Echinoderes astridae* | 0 | 0 | 0 | 0 | - | 0 | 1 | 0 | 0 | 1 | 0 | 0 | 0 | 0 | 0 | 1 | 1 | 1 | 1 | 0 | 0 |
| *Echinoderes aureus* | 0 | 0 | 0 | 0 | - | 0 | 1 | 0 | 0 | 1 | 0 | 0 | 0 | 0 | 0 | 1 | 1 | 1 | 1 | 0 | 0 |
| *Echinoderes capitatus* | 0 | 0 | 0 | 0 | - | 0 | 1 | 0 | 0 | 1 | 0 | 0 | 0 | 0 | 0 | 1 | 0 | - | 1 | 0 | 0 |
| *Echinoderes dujardinii* | 0 | 0 | 0 | 0 | - | 0 | 1 | 0 | 0 | 1 | 0 | 0 | 0 | 0 | 0 | 1 | 1 | 1 | 1 | 0 | 0 |
| *Echinoderes gerardi* | 0 | 0 | 0 | 0 | - | 0 | 1 | 0 | 0 | 1 | 0 | 0 | 0 | 0 | 0 | 1 | 1 | 1 | 1 | 0 | 0 |
| *Echinoderes horni* | 0 | 0 | 0 | 0 | - | 0 | 1 | 0 | 0 | ? | 0 | 0 | 0 | 0 | 0 | 1 | 1 | 1 | 1 | 0 | 0 |
| *Echinoderes marthae* | 0 | 0 | 0 | 0 | - | 0 | 1 | 0 | 0 | ? | 0 | 0 | 0 | 0 | 0 | 1 | 0 | - | 1 | 0 | 0 |
| *Echinoderes microaperturus* | 0 | 0 | 0 | 0 | - | 0 | 1 | 0 | 0 | 1 | 0 | 0 | 0 | 0 | 0 | 1 | 1 | 1 | 1 | 0 | 0 |
| *Echinoderes sensibilis* | 0 | 0 | 0 | 0 | - | 0 | 1 | 0 | 0 | 1 | 0 | 0 | 0 | 0 | 0 | 1 | 1 | 1 | 1 | 0 | 0 |
| *Echinoderes setiger* | 0 | 0 | 0 | 0 | - | 0 | 1 | 0 | 0 | 1 | 0 | 0 | 0 | 0 | 0 | 1 | 1 | 1 | 1 | 0 | 0 |
| *Echinoderes spinifurca* | 0 | 0 | 0 | 0 | - | 0 | 1 | 0 | 0 | 1 | 0 | 0 | 0 | 0 | 0 | 1 | 1 | 1 | 1 | 0 | 0 |
| *Echinoderes truncatus* | 0 | 0 | 0 | 0 | - | 0 | 1 | 0 | 0 | 1 | 0 | 0 | 0 | 0 | 0 | 1 | 1 | 1 | 1 | 0 | 0 |
| *Echinoderes* sp. 1 | 0 | 0 | 0 | 0 | - | 0 | 1 | 0 | 0 | 1 | 0 | 0 | 0 | 0 | 0 | 1 | 1 | 1 | 1 | 0 | 0 |
| *Fissuroderes sorenseni* | 4 | 0 | 0 | 0 | - | 0 | 1 | 0 | 0 | 1 | 0 | 0 | 0 | 0 | 0 | 1 | 1 | 1 | 1 | 0 | 0 |
| *Fissuroderes thermoi* | 0 | 0 | 0 | 0 | - | 0 | 1 | 0 | 0 | 1 | 0 | 0 | 0 | 0 | 0 | 1 | 1 | 1 | 1 | 0 | 0 |
| *Franciscideres kalenesos* | 3 | 0 | 1 | 0 | - | 0 | 0 | 0 | 0 | 0 | 0 | 0 | 0 | 0 | 0 | 0 | 1 | 0 | 1 | 0 | 0 |
| *Kinorhynchus giganteus* | 0 | 0 | 0 | 1 | 1 | 1 | - | 1 | - | - | - | - | 0 | 1 | 0 | 1 | 0 | - | 0 | 0 | 0 |
| *Kinorhynchs yushini* | 0 | 0 | 0 | 1 | 1 | 1 | - | 1 | - | - | - | - | 0 | 1 | 0 | 1 | 0 | - | 0 | 0 | 0 |
| *Meristoderes macracanthus* | 0 | 0 | 0 | 0 | - | 0 | 1 | 0 | 0 | 1 | 0 | 0 | 0 | 0 | 0 | 1 | 1 | 1 | 1 | 0 | 0 |
| *Meristoderes* sp. | 0 | 0 | 0 | 0 | - | 0 | 1 | 0 | 0 | 1 | 0 | 0 | 0 | 0 | 0 | 1 | 1 | 1 | 1 | 0 | 0 |
| *Mixtophyes abyssalis* | 1 | 0 | 0 | 1 | 0 | 1 | - | 1 | - | - | - | - | 0 | 0 | 0 | 1 | 0 | - | 1 | 0 | 0 |
| *Neocentrophyes intermedius* | 1 | 0 | 0 | 1 | 0 | 1 | - | 1 | - | - | - | - | 0 | ? | ? | ? | ? | - | 0 | 0 | 1 |
| *Neocentrophyes satyai* | 1 | 0 | 0 | 1 | 0 | 1 | - | 1 | - | - | - | - | 0 | 0 | 0 | 1 | ? | - | 0 | 0 | 1 |
| *New Genus* | 3 | 0 | 1 | 0 | - | 0 | 0 | 0 | 0 | 0 | 0 | 0 | 0 | 0 | 0 | 0 | 1 | 0 | 1 | 0 | 0 |
| *Paracentrophyes anurus* | 1 | 0 | 0 | 1 | 0 | 1 | - | 1 | - | - | - | - | 0 | 0 | 0 | 1 | 0 | - | 1 | 0 | 0 |
| *Paracentrophyes quadridentatus* | 1 | 0 | 0 | 1 | 0 | 1 | - | 1 | - | - | - | - | 0 | 0 | 0 | 1 | 0 | - | 1 | 0 | 1 |
| *Polacanthoderes martinezi* | 0 | 0 | 0 | 0 | - | 0 | 1 | 0 | 0 | 1 | 0 | 0 | 0 | 0 | 0 | 1 | 1 | 1 | 1 | 0 | 0 |
| *Pycnophyes communis* | 0 | 0 | 0 | 1 | 1 | 1 | - | 1 | - | - | - | - | 0 | 1 | 0 | 1 | 0 | - | 1 | 0 | 0 |
| *Pycnophyes dentatus* | 0 | 0 | 0 | 1 | 1 | 1 | - | 1 | - | - | - | - | 0 | 1 | 0 | 1 | 0 | - | 1 | 0 | 0 |
| *Pycnophyes greenlandicus* | 0 | 0 | 0 | 1 | 1 | 1 | - | 1 | - | - | - | - | 0 | 1 | 0 | 1 | 0 | - | 1 | 0 | 0 |
| *Pycnophyes kielensis* | 0 | 0 | 0 | 1 | 1 | 1 | - | 1 | - | - | - | - | 0 | 1 | 0 | 1 | 0 | - | 1 | 0 | 0 |
| *Pycnophyes oshoroensis* | 0 | 0 | 0 | 1 | 1 | 1 | - | 1 | - | - | - | - | 0 | 1 | 0 | 1 | 0 | - | 1 | 0 | 0 |
| *Pycnophyes robustus* | 0 | 0 | 0 | 1 | 1 | 1 | - | 1 | - | - | - | - | 0 | 1 | 0 | 1 | 0 | - | 1 | 0 | 0 |
| *Pycnophyes tubuliferus* | 0 | 0 | 0 | 1 | 1 | 1 | - | 1 | - | - | - | - | 0 | 1 | 0 | 1 | 0 | - | 1 | 0 | 0 |
| *Pycnophyes zelinkaei* | 0 | 0 | 0 | 1 | 1 | 1 | - | 1 | - | - | - | - | 0 | 1 | 0 | 1 | 0 | - | 1 | 0 | 0 |
| *Semnoderes armiger* (Italy) | 0 | 0 | 1 | 0 | - | 0 | 0 | 0 | 0 | 0 | 0 | 0 | 1 | 0 | 0 | 0 | 1 | 0 | 1 | 1 | 0 |
| *Semnoderes armiger* (Norway) | 0 | 0 | 1 | 0 | - | 0 | 0 | 0 | 0 | 0 | 0 | 0 | 1 | 0 | 0 | 0 | 1 | 0 | 1 | 1 | 0 |
| *Sphenoderes poseidon* | 0 | 0 | 1 | 0 | - | 0 | 0 | 0 | 0 | 0 | 0 | 0 | 1 | 0 | 0 | 0 | 1 | 0 | 1 | 1 | 0 |
| *Triodontoderes anulap* | 2 | 0 | 1 | 0 | - | 0 | 0 | 0 | 0 | 0 | 0 | 0 | 1 | 0 | 1 | 0 | 1 | 0 | 1 | 1 | 0 |
| *Tubulideres seminoli* | 0 | 0 | 1 | 0 | - | 0 | 0 | 0 | 0 | 0 | 0 | 0 | 0 | 0 | 1 | 0 | 1 | 0 | 1 | 1 | 0 |
| *Wollunquaderes majkenae* | 0 | 0 | 1 | 0 | - | 0 | 0 | 0 | 0 | 0 | 0 | 0 | 1 | 0 | 1 | 0 | 1 | 0 | 1 | 1 | 0 |
| *Zelinkaderes brightae* | 2 | 0 | 1 | 0 | - | 0 | 0 | 0 | 0 | 0 | 0 | 0 | 1 | 0 | 1 | 0 | 1 | 0 | 1 | 1 | 0 |
| *Zelinkaderes klepali* | 2 | 0 | 1 | 0 | - | 0 | 0 | 0 | 0 | 0 | 0 | 0 | 1 | 0 | 1 | 0 | 1 | 0 | 1 | 1 | 0 |
| *Zelinkaderes yong* | 2 | 0 | 1 | 0 | - | 0 | 0 | 0 | 0 | 0 | 0 | 0 | 1 | 0 | 1 | 0 | 1 | 0 | 1 | 1 | 0 |
| *Zelinkaderes* sp. 1 | 2 | 0 | 1 | 0 | - | 0 | 0 | 0 | 0 | 0 | 0 | 0 | 1 | 0 | ? | ? | ? | ? | 1 | 1 | 0 |

The morphological character matrix. Characters 16^*^, 17^*^ and 19^*^ are treated as ordered. Other characters are unordered. The characters are listed in Appendix 1 in Supporting Information Files.
